# Therapeutic Failure in Invasive Fungal Infections: Beyond Antifungal Resistance—A Narrative Review

**DOI:** 10.3390/jof12080596

**Published:** 2026-08-11

**Authors:** Pilar Rivas-Pinedo, José Millán Oñate Gutiérrez

**Affiliations:** 1 Medical and Diagnostic Mycology Group, Department of Microbiology, Faculty of Medicine, Universidad Nacional de Colombia, Bogotá 111321, Colombia; 2 One Health Research Group for Infectious Diseases, Keralty, Department of Internal Medicine, Clínica Colsanitas S.A., Clínica Sebastián de Belalcázar, Cali 760045, Colombia; 3 Clinical Research and Education Group (GIEDCO), Clínica de Occidente, Cali 760035, Colombia

**Keywords:** invasive fungal infections, antifungal treatment failure, antifungal resistance, mycological diagnosis, antifungal pharmacology, source control, antifungal stewardship, treatment optimization

## Abstract

Invasive fungal infections (IFIs) are associated with high morbidity and mortality, particularly in immunocompromised or critically ill patients and in those with complex comorbidities. Despite advances in mycological diagnosis and antifungal therapy, outcomes remain poor in a significant proportion of patients. In clinical practice, a poor response is often attributed to microbiological resistance. This interpretation may lead to empirical treatment escalation, combination therapy without a clear indication, increased toxicity, clinically relevant drug–drug interactions, and inefficient use of healthcare resources. However, therapeutic failure rarely results from a single mechanism. More often, it reflects the interaction among host vulnerability, pathogen characteristics, delayed, incomplete, or incorrect diagnosis, insufficient antifungal exposure, and inadequate source control. This narrative review presents a structured clinical approach to evaluating suboptimal responses in IFIs without attributing them solely to antifungal resistance. It examines cross-cutting determinants and specific patterns associated with invasive aspergillosis, invasive candidiasis and persistent candidemia, cryptococcal meningitis, mucormycosis, infections caused by rare filamentous fungi, and disseminated endemic mycoses. It also presents clinical algorithms, checklists, and potential quality indicators to distinguish true from apparent failure, identify modifiable factors, and support transparent clinical decision-making within antifungal stewardship programs. The proposed framework supports the assessment of non-response through systematic diagnostic and therapeutic reassessment, timely source control, and identification of modifiable gaps in care.

## 1. Introduction

### 1.1. Magnitude of the Problem and Persistence of Therapeutic Failure

Invasive fungal infections (IFIs) remain a significant cause of morbidity and mortality among immunocompromised and critically ill patients, as well as patients with complex comorbidities. Global estimates and contemporary clinical series confirm their substantial impact, with particularly poor outcomes associated with invasive aspergillosis (IA), invasive candidiasis (IC), cryptococcal meningitis (CM), mucormycosis, disseminated endemic mycoses, and infections caused by rare or emerging filamentous fungi [1,2,3,4,5]. The clinical burden has become increasingly evident with the growth of vulnerable populations, the emergence of resistant or difficult-to-identify species, and persistent gaps in access to specialized mycological diagnostics, epidemiological surveillance, and timely antifungal treatment [1,2]. The clinical challenge extends beyond the availability of new antifungal agents and includes the early recognition of a suboptimal response and the identification of potentially modifiable factors.

In clinical practice, a lack of response may be attributed too readily to antifungal resistance. Such an interpretation can lead to empirical treatment escalation, combination regimens without a clear indication, prolonged exposure to broad-spectrum antifungals, increased toxicity, and inefficient use of healthcare resources. However, treatment failure in IFIs is rarely explained by resistance alone. Instead, it more often reflects the combined influence of host vulnerability, pathogen characteristics, diagnostic limitations, insufficient antifungal exposure, inadequate source control, and gaps in the care process [6,7,8,9,10,11,12,13].

Attributing a poor response solely to antifungal failure or resistance can divert attention from interventions with greater clinical impact, such as reviewing and broadening the diagnosis, reassessing the causative pathogen, optimizing antifungal exposure, reducing reversible immunosuppression, removing infected devices, draining fluid collections, and undertaking timely surgical intervention when indicated [6,7,8,14,15].

### 1.2. Antifungal Therapeutic Failure: A Concept Broader than Resistance

Antifungal treatment failure may manifest as the absence of the expected clinical, microbiological, or radiological response; persistent fever or clinical deterioration; progression of existing lesions; persistent fungemia; delayed microbiological clearance; early relapse; or the development of new sites of infection. None of these manifestations individually confirms in vitro antifungal resistance [6,8,9,16].

Factors contributing to an inadequate response include delayed or incomplete diagnosis, a high initial fungal burden, absent or limited immune recovery, inadequate antifungal exposure at the site of infection, and inadequate source control. This distinction is particularly relevant in IA; candidemia with persistent intravascular or deep-seated foci; CM with a high fungal burden or uncontrolled intracranial pressure (ICP); mucormycosis with extensive tissue necrosis; and infections caused by rare molds with unpredictable susceptibility profiles or limited therapeutic evidence [4,5,6,8,14,15,17,18,19,20,21,22].

Within this framework, antifungal resistance is one possible determinant of treatment failure, but it should not be assumed to be the primary explanation. The clinical significance of resistance should be assessed as part of a broader diagnostic and therapeutic reassessment that considers the host’s condition, the clinical syndrome, antifungal exposure, and source control [6,7,8,9,23].

### 1.3. Clinical Relevance and Consequences of Therapeutic Failure

Antifungal treatment failure is associated with persistent infection, prolonged hospitalization, repeated diagnostic testing, greater antifungal exposure, toxicity, rising healthcare costs, and successive treatment changes that fail to address the underlying cause of the poor response [6,7,8,11,12,13,24,25].

Within antifungal stewardship programs (AFSPs), treatment failure can be used as a sentinel event to trigger a structured review of the diagnostic and therapeutic process. Such a review should consider diagnostic certainty, treatment timeliness, the activity of the antifungal agent against the identified pathogen, the systemic exposure achieved, source control, and the host’s capacity to respond [24,25,26,27,28,29,30]. Whenever the clinical situation allows, modifiable determinants should be identified before treatment is changed or intensified [6,8,24,26,27,28,30].

### 1.4. Rationale and Objectives

Although disease-specific guidelines are available for the treatment of IFIs, a practical gap remains in the cross-cutting reassessment of patients with a suboptimal response that cannot be explained solely by the initially identified pathogen or clinical syndrome. Guidelines are typically organized by disease entity—aspergillosis, candidiasis, cryptococcosis, mucormycosis, rare mold infections, or endemic mycoses—whereas evaluating a suboptimal response requires simultaneous consideration of the host, pathogen, diagnosis, antifungal exposure, and source control [4,5,14,15,17,19,20,25].

The aim of this narrative review is to present a practical approach to evaluating suboptimal responses in IFIs, distinguishing true from apparent treatment failure and placing antifungal resistance within a broader diagnostic and therapeutic framework. The review examines key determinants related to the host, pathogen, diagnosis, antifungal exposure, and source control, together with common patterns of poor response in IA, IC—particularly persistent candidemia—mucormycosis, infections caused by rare filamentous fungi, and disseminated endemic mycoses.

This review is intended to complement, rather than replace, disease-specific guidelines by providing a structured clinical approach for patients with a suboptimal response to antifungal treatment. The proposed algorithms and checklists are designed to support clinical decision-making, limit unnecessary empirical escalation, and incorporate reassessment of therapeutic failure into antifungal stewardship practice.

### 1.5. Search Strategy and Narrative Synthesis

This narrative review was informed by searches of PubMed/MEDLINE, Scopus, and Web of Science, complemented by manual screening of the reference lists of relevant publications. Publications from 2000 through March 2026 were considered, together with earlier seminal studies whose clinical or conceptual contribution remained relevant. The search strategy combined controlled vocabulary and free-text terms related to invasive fungal infections, therapeutic failure, suboptimal clinical response, antifungal resistance, pharmacokinetics and pharmacodynamics, therapeutic drug monitoring, antifungal stewardship, source control, invasive candidiasis, aspergillosis, cryptococcosis, mucormycosis, endemic mycoses, and infections caused by rare molds.

Priority was given to international clinical practice guidelines, scientific society consensus statements, position papers, systematic reviews, meta-analyses, multicenter studies, and clinically relevant cohort studies. Final selection was based on thematic relevance, clinical applicability, recency, and, where appropriate, the historical clinical or conceptual significance of the publication. Findings were integrated through a qualitative narrative synthesis.

## 2. Definition and Classification of Antifungal Therapeutic Failure

Evaluating a poor response to antifungal treatment requires distinguishing true disease progression from other clinical scenarios, including an expected slow response, host-related deterioration, insufficient antifungal exposure, breakthrough IFI, or microbiological resistance. This differentiation is clinically relevant because the clinical, microbiological, and radiological course of IFIs varies according to the pathogen, clinical syndrome, initial fungal burden, immune status, antifungal exposure, and timing of source control [6,8,9,16,22].

### 2.1. Clinical Variability of the Concept of Therapeutic Failure

In clinical practice, the term “treatment failure” encompasses heterogeneous manifestations, including persistent fever, radiological progression, persistent fungemia, failure of microbiological clearance, relapse, or the development of a new IFI during antifungal prophylaxis or treatment. This variability complicates comparisons across studies, the interpretation of outcomes, and the consistency of therapeutic decision-making [6,9,16,22].

The absence of a clear operational definition may lead clinicians to attribute a poor response prematurely to antifungal resistance, although treatment failure and microbiological resistance are not equivalent. Microbiological resistance accounts for some cases, particularly in specific epidemiological settings or after antifungal exposure that exerts selective pressure. In many cases, however, an unfavorable outcome reflects multiple contributing factors, including delayed diagnosis, high fungal burden, persistent immunosuppression, suboptimal antifungal exposure, and inadequate source control [6,9,22,31]. A clinically useful definition should identify when the observed course warrants structured diagnostic and therapeutic reassessment [6,18,19].

### 2.2. Antifungal Therapeutic Failure: Operational Definition

In this review, antifungal treatment failure is defined as the absence of the expected clinical, microbiological, and/or radiological response, or as disease progression during a regimen initially considered appropriate in terms of antifungal selection, dosage, route of administration, timing of initiation, and duration, based on the available evidence and the patient’s clinical context [6,9,17,19,20,21].

This definition must be applied dynamically and in relation to the antifungal exposure actually achieved. Therapeutic failure does not present uniformly across candidemia, invasive pulmonary aspergillosis, CM, and mucormycosis. A regimen cannot be considered adequate solely because the selected antifungal is recommended in guidelines or consensus statements; antifungal exposure, drug interactions, penetration at the site of infection, and source control must also be considered. Moreover, because some syndromes require several days or weeks before objective improvement becomes evident, assessment performed too early may lead to unnecessary treatment changes [8,9,14,17,18,20,21,26,27,32].

The therapeutic response should be interpreted within a clinically appropriate timeframe for the specific syndrome. Persistent fever, a positive biomarker, or the absence of early radiological improvement do not, in isolation, constitute sufficient evidence of therapeutic failure. Classification should integrate the clinical course, microbiological clearance, biomarker trends, imaging findings, achieved antifungal exposure, and the feasibility of source control [6,14,15,18,19,20,32].

Therapeutic failure must also be distinguished from breakthrough IFI, defined as an IFI that develops during antifungal exposure for prophylaxis or treatment. Although both scenarios can coexist, they are not equivalent. A breakthrough IFI may result from a pathogen outside the spectrum of the administered antifungal, suboptimal exposure, resistance, selection of a less susceptible species under antifungal pressure, or host-related factors. Its evaluation requires focused diagnostic and therapeutic reassessment rather than an automatic assumption of acquired resistance [9,22,33].

### 2.3. Temporal Patterns of Antifungal Therapeutic Failure

The timing of a poor response can guide the initial reassessment. Although temporal classification does not replace individualized clinical evaluation, it can help organize the probable causes according to whether the patient shows an initial lack of improvement, persistent clinical or microbiological abnormalities, or deterioration after an apparently favorable response [6,8,22].

#### 2.3.1. Primary Failure

Primary failure refers to the absence of the expected clinical, microbiological, and/or radiological response during the initial phase of an antifungal regimen initially considered appropriate. The timeframe for recognizing primary failure varies according to the clinical syndrome, pathogen, response domain, and host condition; therefore, a single interval cannot be applied to all IFIs. In persistent candidemia, for example, failure of early microbiological clearance has different implications than initial radiological progression in IA, in which imaging findings may evolve more slowly or even worsen transiently during immune recovery [14,18,19,21,32].

This pattern should prompt consideration of an incorrect or incomplete diagnosis, a high fungal burden, insufficient antifungal exposure, profound or persistent immunosuppression, and inadequate source control. Antifungal resistance should also be considered, but it should not be assumed to be the primary explanation before these potentially correctable factors have been evaluated [6,8,14,18,22].

#### 2.3.2. Secondary Failure

Secondary failure refers to the loss of an initially favorable response, followed by relapse or clinical, microbiological, and/or radiological progression. This pattern may be associated with inadequate source control, persistence of colonized or infected devices, acquired antifungal resistance, drug interactions, altered absorption or metabolism, treatment interruptions, relapse within an anatomical compartment with limited drug penetration, or immune reconstitution–related phenomena [6,9,20,22,33,34].

The distinction between primary and secondary failure helps define reassessment priorities. In primary failure, particular attention should be given to confirming the diagnosis, the appropriateness of the selected antifungal, and the exposure achieved during the initial treatment phase. In secondary failure, the assessment should focus on persistent foci or relapse at an anatomical site, adherence, pharmacokinetic changes, treatment interruptions, and pathogen selection under antifungal pressure [6,9,22,34].

### 2.4. Dimensions of the Therapeutic Response

In addition to the timing of a poor response, assessment can be organized according to the predominant domain affected. Distinguishing clinical non-response, microbiological persistence, and radiological progression reduces the risk of modifying treatment on the basis of a single parameter [6,16,35].

#### 2.4.1. Clinical Failure

Clinical failure may present as persistence or progression of signs and symptoms attributable to the IFI, including persistent fever, respiratory deterioration, neurological involvement, hemodynamic instability, or new evidence of dissemination. However, these findings do not necessarily indicate progression of the fungal infection, as they may also result from coinfections, residual inflammation, treatment-related toxicity, progression of the underlying disease, or immune reconstitution–related phenomena. Determining whether these findings are attributable to the IFI requires integration of the clinical course with microbiological, radiological, and pharmacological findings, together with the patient’s immune status [6,11,13,20,34,35].

#### 2.4.2. Microbiological Failure

Microbiological failure includes persistent positive fungal cultures, persistent fungemia, or failure to clear the pathogen from clinically relevant samples. In candidemia, persistent positive blood cultures should prompt evaluation for intravascular or deep-seated foci, retained infected devices, and confirmation of the species and its antifungal susceptibility [14,21]. In CM, microbiological clearance may be influenced by the initial fungal burden, the activity of the induction regimen, and the antifungal exposure achieved. In IA, galactomannan (GM) trends should be interpreted together with the clinical and radiological response and with prior or concomitant antifungal exposure [17,18,19].

The persistence of a positive biomarker does not necessarily equate to microbiological failure. Some biomarkers may remain detectable due to residual antigen release, analytical variability, or interferences unrelated to an active fungal infection. Interpreting these biomarkers in isolation may overestimate persistent infection and lead to unnecessary treatment changes [5,19,35,36,37,38].

#### 2.4.3. Radiological Failure

Radiological failure refers to the absence of improvement or progression of lesions on imaging studies. Interpretation requires caution because radiological improvement may lag behind the clinical or microbiological response, and the appropriate imaging modality and expected response kinetics vary according to the clinical syndrome. In hepatosplenic candidiasis, ultrasound and computed tomography may help identify and follow hepatosplenic lesions, although radiological abnormalities may persist despite clinical improvement. In IA, serial chest CT findings should be interpreted according to the timing of treatment and immune recovery; an early increase in pulmonary lesion size during neutrophil recovery may reflect immune reconstitution–related inflammation rather than true fungal progression [14,18,19,21,32,39].

Radiological changes should be interpreted according to the timing of assessment, clinical syndrome, immune status, antifungal exposure, biomarker trends, and any new clinical findings. An isolated image, especially during the early stages of treatment, is rarely sufficient to establish therapeutic failure [19,32,35,39,40,41].

### 2.5. True Therapeutic Failure Versus Apparent Therapeutic Failure

Distinguishing true from apparent therapeutic failure is essential to avoid premature treatment changes. Apparent failure may occur when a clinical course consistent with the natural history of the IFI is interpreted as non-response, when assessment is performed before a clinically appropriate interval has elapsed, or when findings related to inflammation, treatment toxicity, comorbidities, coinfections, or immune recovery are attributed to progression of the fungal infection [6,9,10,11,12,13,32,34,35,36,39].

Apparent failure may occur in IA with early radiological worsening; in candidemia with an unrecognized deep-seated focus; in CM with persistent inflammation or uncontrolled ICP; and in critically ill patients with multiple alternative causes of clinical deterioration. Misclassifying apparent failure as true therapeutic failure may lead to unnecessary antifungal escalation, increased toxicity, greater selective pressure, and delays in interventions directed at the underlying cause, such as confirming the diagnosis, optimizing antifungal exposure, or achieving source control [7,10,12,13,20,21,23,32,34,35,42].

### 2.6. Practical Usefulness of the Classification

The proposed classification provides a practical structure for interpreting a poor response and organizing clinical reassessment. Distinguishing primary, secondary, and apparent failure from breakthrough IFI, while also considering the clinical, microbiological, and radiological domains of response, helps place individual findings in context and prioritize possible diagnostic and therapeutic explanations. Key considerations include diagnostic certainty, pathogen identification, antifungal exposure, source control, and the host’s capacity to respond [6,8,9,33,35].

The classification also provides the basis for examining host-, pathogen-, diagnostic-, pharmacological-, and source control–related determinants of therapeutic failure.

Table 1 summarizes the operational classification of antifungal therapeutic failure in IFIs.

## 3. Host Determinants in Antifungal Treatment Failure

The in vitro activity of an antifungal agent against the pathogen does not, by itself, explain the clinical response in IFIs. Host-related determinants influence the initial fungal burden, rate of disease progression, ability to contain tissue invasion, intensity of the inflammatory response, and treatment tolerance. Therefore, these determinants should be incorporated systematically into the clinical reassessment of a poor response, particularly in immunocompromised or critically ill patients, transplant recipients, and patients with significant metabolic comorbidities [3,33,44,45,46].

### 3.1. The Host as a Central Determinant of the Outcome

In IFIs, outcomes depend substantially on the host’s ability to contain fungal invasion. Even when a guideline-recommended antifungal regimen is administered, profound immunosuppression, organ dysfunction, physiological derangements associated with critical illness, or the inability to reverse predisposing factors may limit infection control [3,9,17,19,45,46].

The clinical course may differ even among patients infected with the same pathogen, with a similar anatomical site involved and receiving comparable antifungal regimens. A poor response does not necessarily indicate failure of the antifungal regimen; rather, it may reflect the combined influence of infection aggressiveness, fungal burden, the antifungal exposure achieved, and the host’s immunological and physiological capacity to control the disease [3,22,46,47,48,49].

Host assessment should extend beyond the identification of risk factors to include the severity of the immune defect, its predominant mechanism, expected duration, and potential reversibility. This assessment helps distinguish a slow response consistent with the expected clinical course from progression driven by persistent immunosuppression or apparent failure associated with immune recovery or a paradoxical inflammatory response [3,44,45,46,48,49]. It also supports realistic therapeutic expectations and helps prioritize interventions directed at modifiable factors.

### 3.2. Deep and Persistent Immunosuppression

Deep and sustained immunosuppression alters not only the risk of developing an IFI but also its clinical presentation, the expression of inflammatory signs, the rate of response, and the likelihood of progression or relapse. In patients with hematologic malignancies, transplant recipients, and individuals exposed to corticosteroids, other immunosuppressants, immunomodulatory therapies, or targeted treatments, the clinical course should be interpreted according to the magnitude, duration, and reversibility of the immune deficit [3,8,17,19,44,45,46].

#### 3.2.1. Neutropenia

Profound and prolonged neutropenia is among the factors most consistently associated with poor outcomes in IFIs, particularly IA and other infections caused by filamentous fungi. Neutrophil recovery is closely linked to infection control, whereas persistent neutropenia may limit the clinical response even when the pathogen is susceptible and the antifungal regimen is considered appropriate [18,19,50,51].

In the absence of hematologic recovery, antifungal treatment may partially contain disease progression but not achieve complete infection control. Where feasible, adjunctive measures include reducing immunosuppression, promoting or facilitating neutrophil recovery, and achieving source control. These interventions should be accompanied by clinical, microbiological, and radiological monitoring tailored to the specific syndrome. Transient radiological worsening may also occur during neutrophil recovery in patients with IA and may be misinterpreted as treatment failure if the immune and temporal context is not considered [18,39,41,50,51,52].

#### 3.2.2. Transplantation and Immunosuppressive Therapies

In transplant recipients, a poor response may be influenced by the cumulative intensity of immunosuppression, graft type, time since transplantation, episodes of rejection, exposure to corticosteroids or other immunosuppressants, and the feasibility of reducing these therapies without compromising graft function. In this context, IFI progression does not necessarily indicate resistance or inappropriate antifungal selection; it may instead reflect a persistent immune defect that limits infection containment [3,19,53,54].

Reassessment should include the entire immunosuppressive regimen, including its intensity and duration, relevant drug interactions, and the feasibility of dose reduction, modification, or discontinuation. This review is particularly important when infection progresses despite an active antifungal agent, because progression driven by persistent immunosuppression may be misattributed to antifungal failure. Decisions should balance the potential benefit of facilitating immune recovery against the risks of rejection, graft-versus-host disease, and other complications arising from modifications to immunosuppressive therapy [3,19,46,47,49].

#### 3.2.3. Corticosteroids, Targeted Therapies, and Non-Transplant-Related Immunomodulation

Outside transplantation, the increasing use of systemic corticosteroids, biologic therapies, kinase inhibitors, and other immunomodulators has broadened the population at risk for IFIs and poor treatment outcomes. Although these patients may not fit the classic risk profiles associated with neutropenia or transplantation, some therapies substantially alter cellular immunity, phagocytic function, or the inflammatory response according to their mechanism of action, intensity, and duration [3,19,46,54,55].

In this population, reassessment should identify all immunomodulatory therapies administered, their mechanisms of action, the magnitude and duration of their effects, potential drug interactions, and the feasibility of dose reduction or discontinuation. If these factors are not considered, progression driven by a persistent immune defect may be incorrectly attributed to antifungal resistance or primary treatment failure [3,53,54,55,56,57].

### 3.3. Critically Ill Patients and Organ Dysfunction

In critically ill patients, sepsis, shock, hypoalbuminemia, an increased volume of distribution, renal or hepatic dysfunction, extracorporeal life support (ECLS), enteral nutrition, polypharmacy, and rapidly changing physiology can substantially alter antifungal pharmacokinetics and exposure. Consequently, standard dosing may result in subtherapeutic or potentially toxic antifungal concentrations [47,49,58,59]. Pharmacokinetic studies in critically ill patients have shown inconsistent attainment of pharmacokinetic/pharmacodynamic (PK/PD) targets, even with currently recommended regimens. These findings support individualized dosing and the use of therapeutic drug monitoring (TDM) when clinically appropriate [47,59,60,61].

These considerations are particularly important for antifungal agents with high interindividual pharmacokinetic variability, a narrow therapeutic index, or clinically relevant drug–drug interactions. A poor response may reflect insufficient systemic exposure or inadequate drug concentrations at the site of infection rather than microbiological resistance. Reassessment should include hemodynamic status, renal and hepatic function, extracorporeal support, drug–drug interactions, and TDM when clinically indicated and available [49,58,59,60,62].

### 3.4. Metabolic and Structural Comorbidities

Metabolic and structural comorbidities influence both the risk of developing an IFI and the response to treatment. Their importance is particularly evident in mucormycosis, complicated IC, device-associated infections, and infections involving deep-seated foci. In these settings, control of the underlying condition may be as important as antifungal selection, because pharmacological treatment alone cannot correct the anatomical, metabolic, or vascular abnormalities that promote persistent infection [15,63,64,65].

#### 3.4.1. Diabetes Mellitus

Diabetes mellitus, particularly when accompanied by persistent hyperglycemia, ketoacidosis, or metabolic acidosis, is a recognized determinant of disease severity and poor outcomes in mucormycosis. Impaired phagocytic function, increased free iron availability under acidic conditions, and vascular compromise facilitate tissue invasion, necrosis, and rapid progression. Metabolic control and correction of acidosis are essential components of treatment, together with prompt initiation of an active antifungal agent and timely surgical debridement when indicated and feasible [15,63,66].

In mucormycosis and other IFIs associated with uncontrolled diabetes, a poor response should not be attributed solely to inadequate antifungal activity. Persistent hyperglycemia, acidosis, necrotic tissue, and vascular compromise may limit the response even when the antifungal regimen is considered appropriate [15,63,67].

#### 3.4.2. Renal and Hepatic Dysfunction

Renal and hepatic dysfunction influence antifungal selection, dosing, safety, and treatment continuity. Renal impairment may limit the use of certain formulations, increase toxicity risk, alter exposure to specific antifungal agents, or increase the potential for excipient-related adverse effects. Hepatic dysfunction may alter azole metabolism, increase the risk of hepatotoxicity, and require dose adjustment, treatment interruption, or premature modification of the regimen. In both settings, toxicity may be mistaken for infection-related clinical deterioration or may require treatment interruption, thereby compromising the therapeutic response [58,59,62,64].

Reassessment of a poor response in patients with renal or hepatic dysfunction should consider the dose, dosing interval, route of administration, changes in organ function, drug–drug interactions, cumulative toxicity, and the potential role of TDM. In some cases, a poor response does not reflect insufficient antifungal activity but rather difficulties in maintaining safe and effective exposure for the duration required to control the infection [47,49,58,62].

### 3.5. Immune Reconstitution and Apparent Failure

In certain clinical settings, rapid or partial recovery of immune function may mimic treatment failure. This phenomenon has been described primarily in people with HIV after initiation of antiretroviral therapy (ART), transplant recipients following reduction of immunosuppression, patients with hematologic malignancies during neutrophil recovery, and other settings in which an underlying immune defect begins to reverse. Transient clinical or radiological deterioration may then occur despite a favorable microbiological response or partial control of the infection [34,68,69]. Immune reconstitution inflammatory syndrome (IRIS) may have different manifestations depending on the pathogen and immune context. It is a recognized cause of paradoxical deterioration that may be mistaken for true progression of the infection [69,70].

Distinguishing true fungal progression from immune reconstitution–related inflammation is essential to avoid unnecessary changes in antifungal therapy. Assessment should integrate the longitudinal clinical course, mycological culture results, biomarker trends, imaging findings, fungal burden, immune status, and response to treatment. When considered in isolation, fever, lesion enlargement, or worsening inflammation may lead clinicians to misclassify a paradoxical immune response as treatment failure [20,34,68,69].

### 3.6. Reversibility of the Host Defect

In addition to identifying the predisposing factor, host assessment should determine whether the underlying defect is reversible, partially reversible, or irreversible. The likelihood of response differs substantially between a patient with transient neutropenia and one with persistent immunosuppression, active hematologic disease, irreversible organ dysfunction, or immunosuppressive therapy that cannot be reduced. This distinction helps define realistic therapeutic expectations and the appropriate intensity of monitoring. It also reduces the risk of classifying a limited response caused by a persistent immune or physiological defect as antifungal treatment failure [3,21,46,49,50].

The reversibility of the host defect is an essential component of structured reassessment. The potential for neutrophil recovery, the feasibility of reducing corticosteroids or other immunosuppressants, and the ability to maintain effective and safe antifungal exposure may be as relevant as antifungal selection. These considerations help tailor treatment and monitoring to the patient’s clinical and prognostic context [3,44,46,59,62].

Collectively, host determinants influence the rate, magnitude, and sustainability of the therapeutic response. Their assessment should be dynamic and integrated with pathogen characteristics, antifungal exposure, diagnostic certainty, and source control, helping to identify potentially modifiable factors before a poor response is attributed to antifungal resistance or failure of the antifungal regimen.

## 4. Pathogen Determinants Associated with Treatment Failure

In addition to host-related factors, diagnostic timing, and antifungal exposure, pathogen characteristics influence the likelihood and rate of response, microbiological clearance, persistence in anatomically difficult-to-treat sites, and the risk of relapse. Key determinants include fungal burden, accurate species-level identification, variability in antifungal susceptibility, intrinsic or acquired resistance, and biofilm-forming capacity. Previous antifungal exposure may also favor the selection of less susceptible or resistant fungal subpopulations [4,6,7,23,31,47,49,71,72,73].

### 4.1. Role of the Pathogen in Therapeutic Failure

Although host- and diagnosis-related factors often influence outcomes, the likelihood and rate of response vary across IFIs. Fungal burden, patterns of tissue invasion, antifungal susceptibility, and persistence within specific anatomical foci may influence the clinical course [4,6,7,49,71,72,73].

Incomplete characterization of the causative pathogen may lead to misinterpretation of a poor response. When a clinically relevant isolate is obtained, microbiological evaluation should include, where feasible, species-level identification and antifungal susceptibility testing, interpreted within the epidemiological context and clinical syndrome. This evaluation is particularly important in IC, aspergillosis with suspected azole resistance, infections caused by rare filamentous fungi, and breakthrough IFIs developing during antifungal prophylaxis or treatment [7,9,23,33,46,73,74].

Interpretation of microbiological findings should consider fungal burden, anatomical location, previous antifungal exposure, the expected susceptibility profile, whether the organism represents colonization or invasive infection, and the presence of persistent foci. The isolation or detection of a fungus does not, by itself, explain a poor response. Its clinical significance depends on its consistency with the clinical syndrome and its integration with diagnostic findings, antifungal exposure, anatomical factors, and host-related determinants [4,6,48,49,71].

### 4.2. Initial Fungal Burden and Response Kinetics

In CM, a high initial fungal burden and delayed microbiological clearance have been associated with poor outcomes and may contribute to an incomplete or delayed response even when the induction regimen is considered appropriate [20,75,76,77].

In IA, deep-seated candidiasis, and other IFIs with extensive tissue involvement, clinical, microbiological, or radiological improvement may take longer to become evident. Persistent biomarker positivity early during treatment, a slow decline in fungal burden, or the absence of early radiological improvement does not necessarily indicate resistance or therapeutic failure. These findings may instead reflect a high initial fungal burden, established tissue damage, a host inflammatory response, or inadequate source control [4,14,15,18,19,49,78,79,80].

Interpreting these early findings as treatment failure may lead to unnecessary treatment changes. Instead, the assessment should consider the time elapsed since treatment initiation, the clinical course and immune status, culture results, biomarker trends, imaging findings, and the possibility of persistent foci. When microbiological persistence is documented, additional considerations include the initial fungal burden, antifungal exposure, drug penetration into the affected anatomical compartment, biofilm formation, resistance, and adequacy of source control [6,14,49,71,75,79,80].

### 4.3. Cryptic Species, Emerging Species, and Misidentification

Molecular methods and high-resolution identification platforms have shown that several clinically relevant fungal genera, including *Aspergillus*, *Cryptococcus*, *Fusarium*, *Scedosporium*, and *Lomentospora*, as well as yeasts formerly classified within the genus *Candida*, contain species and species complexes that differ in epidemiology, virulence, and antifungal susceptibility. Therefore, identification limited to the genus level or based on phenotypic methods with insufficient discriminatory power may fail to distinguish species with reduced susceptibility, intrinsic resistance, or important epidemiological implications, potentially contributing to an unexpectedly poor treatment response [4,81,82,83,84].

Among clinically relevant yeasts associated with greater therapeutic complexity, *Nakaseomyces glabratus* (formerly *Candida glabrata*), *Pichia kudriavzevii* (formerly *Candida krusei*), and *Candidozyma auris* (formerly *Candida auris*) pose important challenges related to identification and antifungal susceptibility. *P. kudriavzevii* is intrinsically resistant to fluconazole (FLC), whereas the challenges associated with *C. auris* also include its ability to persist in healthcare environments, colonize patients, form biofilms, and cause healthcare-associated outbreaks. Accurate identification and timely communication of results are therefore important for clinical management and, in the case of *C. auris*, for infection prevention and control [31,73,81,85,86].

Fluconazole-resistant *Candida parapsilosis* has also emerged as a clinically relevant challenge. Failure to recognize this resistance pattern may result in continued exposure to an inactive or suboptimal agent. Species-level identification and AFST are therefore particularly important in persistent candidemia, breakthrough infection, or an unexpected poor response during fluconazole therapy [21,31].

Similarly, cryptic *Aspergillus* species and rare filamentous fungi, including *Fusarium*, *Scedosporium*, and *Lomentospora*, may exhibit variable or unpredictable antifungal susceptibility profiles. In these settings, accurate taxonomic identification and, where appropriate, antifungal susceptibility testing can support interpretation of a poor treatment response and guide antifungal selection [4,31,82,83,84,87,88,89,90,91,92].

Identification of a cryptic or emerging species, or of a species with reduced susceptibility, does not by itself demonstrate therapeutic failure. Its clinical significance depends on the severity and anatomical site of infection, the antifungal exposure achieved, the clinical course, and the feasibility of source control. Species-level identification is an important component of the assessment but does not replace comprehensive clinical reassessment [4,49,83,86,89,90].

### 4.4. Antifungal Resistance: Real Clinical Impact

Documented antifungal resistance is an important cause of therapeutic failure in specific settings, although its clinical impact varies according to the species, antifungal agent, epidemiological distribution, previous antifungal exposure, and clinical syndrome. Among yeasts, azole and echinocandin resistance is particularly relevant in species such as *N. glabratus* and *C. auris*. Relevant mechanisms include alterations in antifungal targets, overexpression of efflux pumps, and mutations associated with reduced echinocandin susceptibility. Species-level identification and antifungal susceptibility testing may therefore have direct therapeutic implications [7,71,73,82,93].

In *Aspergillus fumigatus*, triazole resistance has important clinical consequences, particularly in regions with a high prevalence of resistant isolates and in patients previously exposed to azoles. Resistance may emerge during azole therapy or be associated with environmental selection resulting from the use of azole compounds [7,31,73,74,88,91].

However, the relationship between in vitro resistance and clinical failure is not absolute. Interpretation of antifungal susceptibility results should consider the species, testing method, available clinical breakpoints or epidemiological cutoff values, the quality and representativeness of the isolate, antifungal exposure at the site of infection, fungal burden, immune status, and source control. The clinical impact of reduced susceptibility varies according to these factors, while an infection caused by a susceptible isolate may still progress because of delayed diagnosis, a persistent uncontrolled source, or uncorrected immunosuppression [6,7,49,71,73].

Antifungal resistance should be interpreted as a clinically relevant finding within a broader reassessment. Its clinical relevance is best determined by integrating species confirmation, the susceptibility profile, antifungal exposure, adherence, drug–drug interactions, anatomical site, and host-related determinants [7,31,49,72,93].

#### 4.4.1. Intrinsic Resistance

Intrinsic resistance is clinically relevant when the identified species has intrinsically reduced susceptibility to specific antifungal agents or when particular agents lack activity against the organism. This issue is particularly important in rare filamentous fungi, including *Lomentospora prolificans* and certain *Scedosporium* and *Fusarium* species, as well as in yeasts with unpredictable susceptibility profiles or identification challenges. In such cases, a poor response may reflect a mismatch between the spectrum of the selected antifungal and the pathogen’s inherent susceptibility profile rather than resistance acquired during treatment [4,7,87,88,94,95].

Adequate taxonomic resolution is essential because grouping these organisms into broad categories, such as “non-*Aspergillus* mold” or “rare filamentous fungus,” may obscure clinically relevant therapeutic differences. When intrinsic resistance is suspected, reassessment should determine whether the administered antifungal adequately covers the suspected or identified pathogen, confirm species-level identification, and include antifungal susceptibility testing when indicated and available. Suspected or confirmed intrinsic resistance may warrant specialist consultation and an individualized therapeutic approach [4,7,87,88,94,95].

#### 4.4.2. Acquired Resistance and Selection Under Antifungal Pressure

Acquired resistance may emerge or be selected during previous or prolonged antifungal exposure, particularly in the setting of prophylaxis, repeated treatment courses, or persistent infection with a high fungal burden. This phenomenon is particularly relevant in triazole-resistant *A. fumigatus*, *N. glabratus* with acquired azole or echinocandin resistance, and *C. auris* isolates resistant to multiple antifungal classes [2,7,23,31,73,85,86,91,93].

In these settings, reassessment of a poor response should include confirmation of species-level identification, antifungal susceptibility testing when indicated and available, and review of previous antifungal exposure. Detection or suspicion of resistance does not exclude other contributing factors, including insufficient antifungal exposure, a persistent or uncontrolled infectious focus, and adverse host-related determinants. Selection of resistant fungal subpopulations may coexist with pharmacokinetic variability and persistent infection within an uncontrolled anatomical focus [1,2,5,6].

### 4.5. Breakthrough IFI and Pathogens Outside the Antifungal Spectrum

A breakthrough IFI is a clinical scenario in which pathogen characteristics, antifungal selective pressure, antifungal exposure, and the depth of immunosuppression may converge during prophylaxis or active treatment. It may develop when the administered antifungal lacks activity against the causative pathogen, has an insufficient spectrum, or does not achieve adequate exposure, as well as when resistance or persistent immune impairment is present. The infection may be caused by a pathogen outside the spectrum of the administered antifungal, a species with intrinsic or acquired resistance, or a rare or emerging fungus [9,22,33].

Detection of a breakthrough IFI does not automatically imply acquired resistance. Reassessment should include confirmation of species-level identification, review of the spectrum of the administered antifungal, antifungal exposure—including TDM when indicated—adherence, drug–drug interactions, local epidemiology, and the possibility of a rare, emerging, or mixed fungal infection. Management may require a change in antifungal class, broader antifungal coverage, optimization of antifungal exposure, or source control, depending on the underlying determinant [9,22,33].

### 4.6. Biofilms, Antifungal Tolerance, and Protected Anatomical Niches

Fungal biofilm formation, particularly on intravascular devices, prostheses, endothelial surfaces, and other foreign materials, can reduce antifungal activity and hinder microbiological eradication. In central venous catheter (CVC)-related candidemia, fungal endocarditis, and other device-associated infections, microbiological persistence often reflects an uncontrolled anatomical reservoir rather than intrinsic lack of activity of the selected antifungal agent [14,21,96,97,98].

Certain anatomical compartments and sites—including the central nervous system (CNS), ocular tissues, bone, endocardial vegetations, and deep-seated abscesses—may limit adequate antifungal exposure and contribute to incomplete responses or relapse. Poor outcomes may result from the combined effects of a high fungal burden, insufficient tissue penetration, biofilm formation, necrosis, anatomical barriers, delayed drainage, debridement, or resection, or an inability to perform these procedures when clinically indicated [21,49,96,97,99,100,101].

### 4.7. Coinfection, Mixed Infection, and Emergence of a New Pathogen During the Clinical Course

In critically ill patients, patients with hematologic malignancies, and transplant recipients, a poor response may result from coinfection, mixed fungal infection, or the emergence of a new pathogen during the clinical course. A patient initially treated for aspergillosis may subsequently develop CVC-associated candidemia, breakthrough mucormycosis during azole exposure, or infection caused by a rare mold outside the spectrum of the initial regimen. Persistent clinical deterioration should not automatically be attributed to failure of therapy directed against the initially identified pathogen. Rare yeasts, including *Saccharomyces* species, should also be considered when the clinical course is not fully explained by the initially identified pathogen [4,22,97,102,103].

When the clinical course is inconsistent with the initial diagnosis, the etiology should be reassessed by repeating cultures, obtaining additional or alternative samples, reviewing imaging studies, and investigating alternative diagnoses and concomitant bacterial, viral, or fungal infections. Such reassessment may reduce anchoring bias and the risk of intensifying antifungal treatment without addressing the underlying cause of clinical deterioration [6,22,33,39].

Microbiological findings should be interpreted in relation to species-level identification, fungal burden, antifungal susceptibility, biofilm-forming capacity, previous antifungal exposure, and the anatomical site of infection. Microbiological reassessment is particularly important during antifungal prophylaxis or treatment, in the presence of persistently positive cultures, radiological progression, new infectious foci, or suspected mixed infection [4,6,9,22,33,96,97,103].

## 5. Pharmacological Determinants of Therapeutic Failure

Among the potentially modifiable determinants of a poor response, antifungal exposure plays a central role. Treatment failure may occur when the administered regimen does not achieve or maintain adequate antifungal exposure, either systemically or at the site of infection, or when toxicity necessitates dose reduction, treatment interruption, or a change in antifungal therapy before infection control is achieved. Although inadequate antifungal exposure differs from microbiological resistance, both mechanisms may coexist and contribute to a poor outcome. Pharmacological determinants are particularly relevant in critically ill patients, transplant recipients, patients with hematologic malignancies, and infections involving anatomical compartments with limited antifungal penetration [10,12,13,19,47,49,58,60,71,101].

### 5.1. Pharmacological Failure as an Underestimated Cause of Poor Response

In some patients, the selected antifungal may be guideline recommended, yet the administered regimen may not achieve the exposure required for an adequate clinical or microbiological response. Potential causes include an insufficient initial dose, omission of a loading dose when indicated, erratic absorption, drug–drug interactions, interindividual pharmacokinetic variability, physiological changes associated with critical illness, and limited penetration into the affected anatomical compartment [10,19,47,49,58,101].

Before treatment is modified, the prescribed regimen should be distinguished from the antifungal exposure actually achieved. Assessment of actual exposure should include the dose, dosing interval, route and formulation, timing of treatment initiation, missed doses or interruptions, adherence, absorption, renal and hepatic function, drug–drug interactions, anatomical site of infection, and TDM when indicated and available. Dose reductions, treatment interruptions, or discontinuation because of toxicity should also be documented [10,48,59,62,104].

Assessment of actual antifungal exposure may identify subtherapeutic concentrations or an insufficient duration of effective therapy that may not be evident from the prescription alone. This assessment may reduce the risk of prematurely attributing a poor response to antifungal resistance, irreversible disease progression, or inadequate intrinsic activity of the selected antifungal [59,60,62].

### 5.2. Errors in Dosing and Selection of the Antifungal Regimen

Selecting an appropriate antifungal regimen involves more than choosing an agent with in vitro activity against the pathogen. It also requires selecting an appropriate dose, dosing interval, formulation, and route of administration; achieving adequate early antifungal exposure; considering the anatomical site of infection; and individualizing therapy based on the patient’s clinical course and pharmacokinetic characteristics. In severe IFIs, suboptimal exposure during the first days of treatment may compromise early infection control, particularly in patients with a high fungal burden, profound immunosuppression, or inadequate source control [59,60,61,62,104,105,106].

#### 5.2.1. Insufficient or Non-Optimized Dose

Suboptimal dosing, particularly during the initial treatment phase, may contribute to primary treatment failure. This issue is particularly relevant for triazoles because plasma exposure may vary substantially among patients. Voriconazole (VRC), posaconazole (POS), and itraconazole (ITC) exhibit substantial pharmacokinetic variability; insufficient antifungal concentrations may reduce the likelihood of response, whereas excessive concentrations increase the risk of toxicity [47,48,49,59,62,104].

Delayed attainment of therapeutic concentrations may create an early period of insufficient exposure, particularly in rapidly progressive IFIs. When appropriate for the selected antifungal and clinical syndrome, a loading dose, initial intravenous administration, assessment of oral absorption, and early TDM-guided dose adjustment may reduce this risk. Reassessment of the regimen should establish whether the antifungal exposure achieved is adequate for the specific clinical syndrome [47,49,59,62,104,105,107].

#### 5.2.2. Inappropriate Selection of the Antifungal Agent for the Syndrome or Site of Infection

In some cases, treatment failure results from selecting an antifungal that is unsuitable for the specific syndrome or anatomical site despite demonstrating in vitro activity against the pathogen. One example is the use of echinocandins as primary therapy for CNS infection, given their generally limited and variable exposure within this compartment. Although high-dose micafungin has been studied in neonates, these findings should not be extrapolated to routine primary treatment of CNS fungal infections across other populations. The management of deep-seated abscesses, endocardial vegetations, and infections involving bone, ocular tissues, or the intra-abdominal cavity should consider tissue penetration, local antifungal exposure, fungal burden, and the feasibility of source control [18,19,99,100,101,108].

These cases reflect a mismatch between the pharmacological properties of the antifungal and the requirements of the infection site rather than microbiological resistance. In vitro activity alone does not establish that an agent is appropriate for a specific anatomical compartment. Reassessment should determine whether the administered regimen achieves sufficient exposure within the affected compartment and whether anatomical barriers limit local antifungal activity. When local exposure is insufficient or uncertain, selecting an agent with better penetration into the affected compartment, changing the formulation, optimizing source control, or seeking specialist consultation may be preferable to relying solely on empirical dose escalation [18,19,58,99,100,101,108,109].

### 5.3. Tissue Penetration and Drug–Compartment Discordance

Adequate plasma exposure does not necessarily ensure effective antifungal concentrations at the site of infection. Within the CNS, cerebrospinal fluid concentrations do not necessarily reflect exposure in the brain parenchyma, and low or undetectable cerebrospinal fluid concentrations should not be interpreted as evidence of absent tissue distribution. Certain anatomical compartments and sites, including the CNS, ocular tissues, bone, endocardial vegetations, deep-seated abscesses, intra-abdominal collections, and necrotic tissue, may limit antifungal penetration and local activity. A poor response may therefore reflect a drug–compartment mismatch rather than microbiological resistance, even when the selected antifungal shows in vitro activity against the pathogen [10,14,99,101,110,111].

Drug–compartment mismatch is particularly relevant in CNS infections, endophthalmitis, intra-abdominal candidiasis (IAC), fungal endocarditis, osteomyelitis, and mucormycosis with extensive necrosis. Pharmacological reassessment should consider both antifungal activity against the pathogen and the exposure that can be achieved at the site of persistent infection. When antifungal penetration is limited, drainage, debridement, removal of infected material, or selection of an agent with more favorable tissue distribution may be clinically more relevant than empirical dose escalation [15,58,100,101,109,110,111].

In IAC, the abdominal cavity represents a complex pharmacological compartment in which local antifungal concentrations may be insufficient and successful treatment depends heavily on adequate source control. This scenario illustrates how pharmacological and anatomical determinants may coexist and jointly contribute to a poor treatment response [100,101,108,109].

### 5.4. Pharmacokinetic Alterations in Critically Ill Patients

Critically ill patients experience pathophysiological changes that may substantially alter the pharmacokinetics, pharmacodynamics, and resulting exposure of antifungal agents. Relevant factors include an increased volume of distribution, hypoalbuminemia, renal or hepatic dysfunction, variable drug clearance, enteral nutrition, shock, renal replacement therapy, extracorporeal membrane oxygenation (ECMO), and polypharmacy [47,58,60,61,104,112]. Augmented renal clearance may further increase FLC elimination and contribute to subtherapeutic exposure despite conventional dosing [59,60].

These factors may result in subtherapeutic antifungal concentrations despite standard dosing or, conversely, excessive exposure and toxicity when drug elimination is substantially reduced. Moreover, plasma concentrations do not always reflect antifungal exposure or activity at the site of infection, particularly in protected anatomical compartments, necrotic tissue, intra-abdominal collections, or biofilm-associated infections. Antifungal dosing should therefore be individualized rather than assumed to be adequate simply because it follows a conventional regimen [47,49,58,60,61,101,112].

Available evidence indicates that conventional dosing does not consistently achieve adequate antifungal exposure in critically ill patients. Multicenter pharmacokinetic studies, including SAFE-ICU, have shown inconsistent attainment of exposure targets. Evidence from patients receiving ECMO also indicates that ECLS may alter the pharmacokinetics of several antifungal agents and increase uncertainty in dose selection [61,113].

TDM may be particularly useful in critically ill patients, although it is not universally available and its results must be interpreted in the appropriate clinical context. Clinical decision-making should consider infection severity, dynamic changes in organ function, the use of ECLS, drug–drug interactions, toxicity risk, and the probability of achieving adequate antifungal exposure at the affected site [60,62,104].

### 5.5. Clinically Relevant Drug–Drug Interactions

Clinically relevant drug–drug interactions are a potentially preventable cause of suboptimal antifungal exposure, toxicity, and treatment interruption or discontinuation. Azoles are particularly relevant because they may act as substrates and inhibitors of cytochrome P450 enzymes and drug transporters, altering both their own exposure and that of concomitantly administered medications. These interactions are particularly important in transplant recipients, patients with hematologic malignancies, and critically ill patients, who frequently receive immunosuppressants, antineoplastic therapy, anticonvulsants, antiarrhythmics, anticoagulants, proton pump inhibitors, and multiple antimicrobial agents [11,13,47,114].

Clinical consequences may include subtherapeutic antifungal concentrations, toxicity requiring dose reduction or treatment discontinuation, and clinically important changes in the exposure of concomitant medications, including calcineurin inhibitors, mammalian target of rapamycin (mTOR) inhibitors, selected antineoplastic agents, and other drugs with a narrow therapeutic index. The medication regimen should be reviewed for clinically relevant interactions when antifungal therapy is initiated, whenever concomitant medications are added, withdrawn, or modified, and when a poor response or unexpected adverse event occurs [10,11,12,13,60,62,104,114].

If drug–drug interactions are not systematically assessed, insufficient antifungal exposure or avoidable toxicity may be misattributed to antifungal resistance or failure of the selected regimen. For this reason, their systematic assessment should form part of routine medication review within AFSPs [24,48,60,62,104,114].

### 5.6. Drug-Related Toxicity, Treatment Interruptions, and Failure to Maintain Antifungal Exposure

Pharmacological treatment failure may also result from the inability to maintain adequate antifungal exposure for the required duration. Nephrotoxicity, hepatotoxicity, neurotoxicity, electrolyte disturbances, gastrointestinal intolerance, QT-interval prolongation, or clinically significant drug–drug interactions may require dose reduction, temporary treatment interruption, or premature modification of the antifungal regimen. These treatment modifications may create periods of insufficient antifungal exposure that are subsequently misinterpreted as inadequate intrinsic activity of the selected agent [10,47,49,59,62,112,114].

Assessment of a poor response should include a detailed reconstruction of antifungal exposure, including the doses administered, missed doses, dose reductions, dosing intervals, changes in formulation or route of administration, treatment interruptions due to toxicity, adherence, drug availability, and the effective duration of active therapy. In some patients, the problem lies not with the selected antifungal agent but with the inability to maintain effective and safe antifungal exposure for the duration required to control the infection [24,49,62,101,112].

This issue is particularly relevant during prolonged treatment, in deep-seated infections, in patients with renal or hepatic dysfunction, and when the therapeutic margin between efficacy and toxicity is narrow. Individualized dose adjustment, TDM when indicated and available, and proactive toxicity monitoring may help maintain more stable antifungal exposure and reduce unnecessary treatment changes [62,104,114].

### 5.7. Therapeutic Drug Monitoring and Optimization of Antifungal Exposure

TDM provides an objective basis for assessing and optimizing antifungal exposure, particularly in patients receiving VRC, POS, ITC, or flucytosine (5-FC). Its primary value lies in identifying subtherapeutic concentrations that may compromise efficacy and elevated concentrations associated with an increased risk of toxicity. For isavuconazole (ISA), routine TDM is not universally recommended, but concentration measurement may be considered in selected patients with an unexpected poor response, suspected altered exposure, clinically relevant drug–drug interactions, toxicity, or other pharmacokinetic concerns. More broadly, TDM may be particularly useful in critically ill patients, patients with hematologic malignancies, transplant recipients, and patients with suspected malabsorption or a poor treatment response. In these settings, TDM can help determine the antifungal exposure achieved and assess its potential contribution to the clinical course [44,47,48,59,60,62,104,107].

However, TDM does not replace comprehensive clinical assessment. A concentration within the recommended target range does not guarantee a favorable response when the diagnosis is incorrect, source control is inadequate, the pathogen is resistant, the fungal burden is high, or antifungal penetration into the affected site is limited. Similarly, a low concentration should be interpreted within the broader clinical context and should not be used as the sole criterion for treatment modification [49,61,62,104].

The availability and turnaround time of TDM vary across centers and may limit its clinical utility. In high-risk patients, limited access to TDM may hinder recognition of subtherapeutic or potentially toxic concentrations and may lead to treatment changes without adequate pharmacological assessment. Clinical practice studies have documented limited use of TDM among hospitalized patients receiving ITC, POS, or VRC, with potential missed opportunities to detect subtherapeutic concentrations or reduce toxicity risk [107]. As part of multidisciplinary reassessment, TDM may help optimize antifungal exposure and improve interpretation of a poor treatment response [24,59,62,104].

Comprehensive review of the dose, dosing interval, route and formulation, absorption, drug–drug interactions, antifungal exposure achieved, toxicity, treatment continuity, penetration into the affected site, and TDM findings can identify potentially correctable pharmacological determinants and clarify their contribution to a poor response.

## 6. Diagnostic Determinants of Therapeutic Failure

An incomplete or delayed diagnosis may compromise the therapeutic response from the outset of antifungal treatment. Establishing the etiology of an IFI rarely depends on a single test and usually requires integration of clinical suspicion, host-related factors, imaging findings, microbiological and histopathological studies, biomarkers, and molecular assays. When a clinically relevant isolate is recovered, species-level identification and antifungal susceptibility testing, when indicated and available, may provide additional information to guide treatment. Delayed sample collection, failure to request or perform relevant tests, and interpretation of results in isolation may delay initiation of active therapy, lead to inappropriate antifungal selection, or create the appearance of therapeutic failure [14,15,16,17,19,21,35,36,37].

### 6.1. The Diagnosis as an Early Determinant of Outcome

Timely diagnosis increases the likelihood that active antifungal therapy can be initiated before the fungal burden and tissue damage progress. Diagnostic delay or incompleteness may postpone treatment and result in selection of an antifungal that is unsuitable for the pathogen, clinical syndrome, or affected anatomical site. This issue is particularly relevant in candidemia, IA, mucormycosis, cryptococcosis, and disseminated histoplasmosis, conditions in which disease progression may be rapid or the fungal burden may already be high at diagnosis [14,15,18,19,20,103,115,116,117,118].

The diagnosis of IFIs is often probabilistic and relies on tests with variable sensitivity, prolonged turnaround times, and results that require interpretation within the clinical context. A single negative result does not exclude an IFI, particularly after previous antifungal exposure, when the circulating fungal burden is low, when disease is confined to tissue, when samples are nonrepresentative, or when the pathogen is difficult to recover in culture [16,36,37,103,115,116,119].

Diagnostic failure should not be understood solely as the absence or unavailability of an appropriate test. It may also represent a process failure extending from initial clinical suspicion and test selection through the interpretation and timely communication of results. Even an appropriate diagnostic test may have limited clinical impact if it is requested late or interpreted without considering the host and clinical syndrome, because under these circumstances it may fail to modify the therapeutic course in a timely manner [36,103,115,116,119].

### 6.2. Diagnostic Delay and Delayed Initiation of Antifungal Treatment

Delayed initiation of appropriate antifungal treatment has been associated with poorer outcomes in candidemia and other IFIs. In candidemia, observational studies have linked delays in administering an active antifungal agent to increased mortality. In mucormycosis, diagnostic delay may permit further angioinvasive progression and extensive tissue necrosis, while reducing the opportunity for early surgical intervention. In IA, delayed clinical suspicion postpones the integration of imaging and biomarkers and, consequently, the initiation of targeted therapy [4,14,15,17,18,19,21,37,117,118,120].

This problem is not always attributable to limited antifungal availability. It is often related to low clinical suspicion, nonspecific manifestations, attribution of deterioration to alternative causes, excessive reliance on early negative results, or delayed access to complementary mycological methods. When treatment begins at an advanced stage, even an otherwise appropriate regimen may not be sufficient to control a high fungal burden or an established anatomical focus [14,15,18,19,117,118].

### 6.3. Limitations of Conventional Methods and Mycological Identification

Conventional methods remain indispensable because they can demonstrate viable fungi, document tissue invasion, and enable subsequent identification and antifungal susceptibility testing. However, their performance depends on specimen quality and timing, fungal burden, prior antifungal exposure, processing conditions, and laboratory expertise [103,119,121,122].

#### 6.3.1. Fungal Cultures

Fungal cultures remain essential because they permit recovery and identification of viable organisms and, when indicated, antifungal susceptibility testing. Nevertheless, their sensitivity is limited in several IFIs, and prolonged growth times may delay therapeutic decisions. A negative culture does not exclude IA, mucormycosis, disseminated histoplasmosis, or an infection confined to tissue that was not adequately sampled [19,103,115,119,121].

In candidemia, blood cultures remain fundamental, although they may require time to become positive and cannot always identify persistent deep-seated foci. In mold infections, cultures may remain negative despite histopathological evidence of tissue invasion. A negative result should therefore not be interpreted in isolation as evidence against an IFI, as this may delay both treatment initiation and collection of a more representative specimen [14,18,19,21,103,119].

#### 6.3.2. Direct Examination and Histopathology

Direct examination can demonstrate fungal structures, whereas histopathology can additionally confirm tissue invasion. These methods are particularly relevant in mucormycosis, IA, and other mold infections, in which evidence of vascular or tissue invasion may immediately alter both the antifungal and surgical management strategy [15,103,119,122,123].

However, histopathology does not always permit species-level identification and may not reliably distinguish among fungi with similar morphological features. Histopathological evaluation also requires tissue sampling, which may not be feasible in critically ill or thrombocytopenic patients or when lesions are located in anatomically difficult-to-access sites. Delays may occur at several stages, including specimen collection, processing, special staining, and reporting. Histopathological findings should therefore be interpreted together with culture results, mycological identification, biomarkers, and molecular assays, where available [15,16,103,119,122,123].

#### 6.3.3. Mycological Identification Using Conventional Methods and Rapid Platforms

Conventional mycological identification—based on macroscopic and microscopic morphological characteristics, biochemical tests, and other phenotypic systems—remains useful but may be insufficient to identify cryptic, emerging, or uncommon species with unpredictable antifungal susceptibility profiles. In yeasts, delayed or incomplete species-level identification may hinder recognition of species associated with antifungal resistance, multidrug resistance, or important epidemiological implications, such as *N. glabratus* and *C. auris*. For filamentous fungi, identification limited to the genus level may prevent recognition of cryptic *Aspergillus* species and rare or emerging molds from genera such as *Fusarium*, *Scedosporium*, and *Lomentospora*, as well as other opportunistic fungi characterized by intrinsic resistance or limited therapeutic evidence [4,23,31,71,74,82,91,92,103,124,125,126].

When MALDI-TOF mass spectrometry supported by up-to-date databases is available, species-level identification of yeasts and selected filamentous fungi can be substantially accelerated. Its performance depends on the breadth and quality of the reference database, the extraction protocol, the representation of cryptic or uncommon species, and laboratory expertise. This is particularly relevant for *C. auris*, because misidentification by phenotypic systems or platforms using outdated databases may have immediate clinical and epidemiological consequences. Rapid identification should be integrated with antifungal susceptibility testing, screening strategies when indicated, and molecular confirmation in complex cases [4,86,103,126,127].

Identification results should be interpreted in relation to the clinical syndrome, anatomical site of infection, and expected antifungal susceptibility profile. When conventional methods yield incomplete results or findings that are inconsistent with the clinical course, higher-resolution techniques—such as MALDI-TOF MS, sequencing, or molecular assays—may be considered, depending on local availability. These techniques may help reduce classification errors and support more precise antifungal selection [23,31,73,74,103,115,121,124,125,126,127].

For species that have undergone recent taxonomic changes, the first mention should include both the updated name and the name most widely recognized in clinical practice to facilitate interpretation and maintain consistency with the previously published literature. This convention is particularly useful for *Nakaseomyces glabratus* (formerly *Candida glabrata*) and *Candidozyma auris* (formerly *Candida auris*), whose historical names remain widely used in clinical practice [2,73,81,91,92].

### 6.4. Biomarkers: Clinical Utility and Limitations of Interpretation

Biomarkers have improved the early diagnosis of several IFIs, but their performance depends on host characteristics, the clinical syndrome, sample type, cutoff value, previous antifungal exposure, fungal burden, and pretest probability. Their diagnostic performance also varies across pathogens, age groups, patient populations, specimen types, and anatomical compartments. When biomarker results are interpreted in isolation, they may lead to misdiagnosis, unnecessary antifungal escalation, or inaccurate assessment of the therapeutic response [16,17,19,36,37,103,128].

A positive or negative biomarker result should be regarded as evidence that modifies diagnostic probability rather than as a substitute for clinical correlation or microbiological confirmation when feasible. Its interpretation should account for the stage of disease, previous antifungal exposure, host characteristics, and the anatomical site of infection [16,17,19,36,37,103,128,129].

#### 6.4.1. Galactomannan

GM testing in serum and bronchoalveolar lavage (BAL) fluid is a useful diagnostic tool for IA, particularly in patients with hematologic malignancies and hematopoietic stem cell transplant recipients. Its performance varies according to host characteristics, sample type, and previous antifungal exposure; therefore, results should be interpreted together with the clinical presentation, imaging findings, and underlying risk factors [17,37,38,78,129,130,131].

Available GM assays include conventional enzyme immunoassays, lateral-flow formats, and automated chemiluminescent methods. Because these platforms differ in analytical characteristics, validated specimen types, cutoffs, and reporting systems, their results are not necessarily interchangeable. When GM is used for longitudinal assessment, serial measurements should preferably be performed using the same assay and specimen type [37,38,129,130,131].

False-negative results may occur in patients receiving mold-active antifungal therapy, in localized disease, or when the circulating antigen burden is low. False-positive results may be associated with analytical factors or specific exposures and require interpretation within the clinical context. Persistent or fluctuating GM values should not be interpreted in isolation as evidence of treatment failure. Serial trends and their relationship to the clinical course and imaging findings are more informative [17,19,35,78,128,130].

#### 6.4.2. β-D-Glucan (BDG)

BDG is a pan-fungal biomarker that serves as a useful diagnostic aid for selected IFIs, including IC and invasive aspergillosis. However, it lacks genus- or species-level specificity and is therefore not informative for all invasive fungal diseases. BDG may yield negative results in mucormycosis and cryptococcosis and may also produce false-positive results because of non-fungal causes or interference associated with medical procedures, biological products, or medical devices [14,37,103,132].

During the reassessment of a poor treatment response, persistent BDG positivity should not be interpreted in isolation as evidence of disease progression. BDG concentrations may decline slowly, and persistently elevated values may reflect ongoing antigen clearance rather than active infection or arise from non-infectious sources. Interpretation should integrate culture results, imaging findings, pathogen-specific biomarkers, the clinical course, and pretest probability [21,36,37,103,132].

#### 6.4.3. Cryptococcal Antigen (CrAg)

CrAg detection is a key diagnostic tool for cryptococcosis, particularly CM and disseminated cryptococcosis. Available platforms include latex agglutination, enzyme immunoassays, lateral flow assays (LFAs), and selected automated methods. LFAs enable rapid CrAg detection in serum, plasma, whole blood, and cerebrospinal fluid (CSF) and are particularly useful in individuals with advanced HIV and other immunocompromised patients [20,103,133,134].

CrAg results should be interpreted in the context of the clinical syndrome and the anatomical compartment being evaluated. High CrAg titers may be associated with a greater fungal burden, but persistently positive titers after treatment do not necessarily indicate microbiological failure. During the monitoring of CM, the clinical course, control of ICP, CSF culture sterilization, and immune status are generally more informative than isolated changes in CrAg titers [20,103,133,134].

#### 6.4.4. *Histoplasma* Antigen

Urine and serum *Histoplasma* antigen detection has facilitated the diagnosis of disseminated histoplasmosis, particularly in individuals with advanced HIV and other forms of immunosuppression. It is particularly valuable when cultures require prolonged incubation or when obtaining tissue samples is not feasible. In endemic regions, timely access to these assays can reduce diagnostic delay and facilitate early treatment initiation [5,135,136,137,138].

In Latin America, where disseminated histoplasmosis may be mistaken for tuberculosis (TB), lymphoma, or other systemic diseases, antigen detection is particularly important in individuals with advanced HIV. Recent reviews of *Histoplasma* antigenuria in people with HIV in Latin America and Africa support its contribution to improving diagnostic access in settings with a high disease burden [5,138,139].

Limitations of *Histoplasma* antigen testing include cross-reactivity with other endemic mycoses, inter-assay variability, lower diagnostic performance in certain localized forms, and unequal regional availability. Results should be interpreted together with the epidemiological history, clinical presentation, imaging findings, culture results, histopathology, and—where applicable—serology or molecular assays. Trends in antigen levels may provide useful information during follow-up but should not be interpreted in isolation to determine treatment response or treatment failure [5,135,136].

### 6.5. Molecular Tests and Complementary Rapid Diagnostic Methods

Molecular tests complement the diagnosis of IFIs, particularly when cultures are negative, histopathology does not permit species-level identification, or the detection of resistance-associated mechanisms is required. These tools include PCR performed on various sample types, sequencing of fungal genomic regions, syndromic panels, detection of resistance-related mutations, and—in selected contexts—metagenomic sequencing. Their main advantages include reducing the time to pathogen detection or identification and increasing diagnostic yield when fungal growth is slow or cultures remain negative [16,17,19,120,140,141,142].

In IA, PCR for *Aspergillus* in blood or BAL fluid can complement GM testing, imaging findings, and host factors, although its performance depends on sample type and quality, assay standardization, fungal burden, and previous antifungal exposure. In mucormycosis, PCR performed on tissue or respiratory samples can be useful when histopathology demonstrates compatible hyphae, but cultures remain negative or fail to identify the causative agent. In IC, molecular tests can accelerate pathogen detection or identification, although their availability, cost, standardization, and clinical validation vary across centers [15,17,21,120,140,142].

Molecular tests can also provide information on resistance mechanisms, such as mutations associated with azole resistance in *A. fumigatus* or clinically relevant genetic alterations in yeasts. However, they cannot universally replace culture or phenotypic antifungal susceptibility testing because they do not always permit recovery of a viable organism, may fail to detect all resistance mechanisms, and require expert interpretation. They are most useful when incorporated into a diagnostic algorithm rather than interpreted in isolation [23,73,74,82,83,93,103,120].

Available evidence indicates that PCR and other nucleic acid–based techniques can improve diagnostic yield and reduce the time to pathogen detection or identification. However, their performance depends on assay standardization, sample selection and quality, pretest probability, contamination control, and integration with culture, histopathology, biomarkers, and imaging findings [103,141,142]. In patients with a poor treatment response, these tools can help correct an incomplete diagnosis or reveal a pathogen not covered by the initial antifungal regimen, a cryptic species, a mixed infection, or an unrecognized resistance mechanism. Their use is particularly valuable in high-risk patients, when tissue is available, or when cultures remain negative despite persistent clinical suspicion—provided that the results can be adequately interpreted [16,74,120,140,142].

### 6.6. Errors in Syndromic Classification

Diagnostic failure may also result from incorrect or incomplete syndromic classification. In aspergillosis, a major challenge is distinguishing respiratory colonization from invasive disease while also avoiding the exclusion of invasive infection solely because cultures are negative. In candidiasis, persistent fungemia may be incorrectly attributed to treatment failure when it is instead caused by a deep-seated focus, a retained infected catheter, or unrecognized endocarditis. In cryptococcosis, neurological deterioration may be attributed to disease progression when the underlying mechanism is uncontrolled ICP or IRIS [14,16,20,21,43,128].

These misinterpretations may lead to inappropriate therapeutic modifications and perpetuate the impression of antifungal treatment failure. Syndromic classification should be dynamically reassessed by integrating information on the anatomical site of infection, host factors, imaging findings, culture results, biomarkers, molecular assays, antifungal susceptibility, and the clinical course over time. This reassessment helps determine whether the syndrome corresponds to the initially suspected IFI and facilitates the evaluation of alternative diagnoses or concomitant processes that may explain the poor treatment response [16,19,20,21,35,43,143,144].

### 6.7. Clinical and Programmatic Impact of Diagnostic Failure

Diagnostic failure may lead to delayed or inappropriate initiation of antifungal treatment, unnecessary use of broad-spectrum antifungal agents, increased toxicity, higher costs, delayed source control, and difficulties in assessing the therapeutic response. It may also prompt empirical antifungal escalation that fails to address the underlying cause of the poor treatment response [24,30,43,48,59,60,63,109,118,143,144].

In patients with a poor treatment response, the diagnosis should be reassessed throughout the clinical course. Integrating clinical suspicion, sample quality and representativeness, species-level mycological identification when feasible, biomarkers, molecular assays, imaging findings, and the clinical course over time may reduce classification errors and guide more specific interventions [16,30,37,43,103,115,120,123,124,128,140,144,145]. From the perspective of AFSPs, episodes of diagnostic failure may serve as warning signals of delays in clinical suspicion, gaps in access to diagnostic testing, communication difficulties between the laboratory and the clinical team, and opportunities to optimize targeted antifungal use [24,25,27,29,30].

Overall, diagnostic determinants influence the entire clinical pathway, from initial suspicion to interpretation of the therapeutic response. Reassessing these determinants helps distinguish ineffective antifungal treatment from an incomplete diagnosis, incorrect syndromic classification, or an unrecognized infection. It also provides a basis for determining whether the priority is to expand the diagnostic evaluation, modify antifungal treatment, or intervene at a persistent infectious focus.

## 7. Control of the Infectious Focus and Therapeutic Failure

In several IFIs, the therapeutic response depends as much on antifungal activity as on effective source control. Persistent deep-seated anatomical foci, infected devices, prosthetic material, necrotic tissue, undrained collections, or biofilms can limit the clinical and microbiological response, even when the antifungal agent is active and antifungal exposure is adequate. Before attributing a poor treatment response to antifungal resistance or pharmacological failure, it is necessary to determine whether there is a persistent reservoir amenable to drainage, debridement, resection, or removal of infected material and to assess the appropriateness and feasibility of the proposed intervention [14,15,21,100].

### 7.1. Control of the Focus as a Determinant of the Therapeutic Response

In some IFIs, antifungal treatment may be insufficient when an anatomical reservoir persists. Necrotic tissue, prosthetic material, infected devices, vegetations, abscesses, and undrained collections may sustain the infection despite antifungal activity and adequate systemic antifungal exposure [6,14,21,98,100,146].

This principle is particularly relevant in mucormycosis, device-associated candidiasis, IAC, fungal endocarditis, and infections involving deep abscesses. In these contexts, antifungal therapy alone may be insufficient and, when indicated and feasible, should be complemented by debridement, drainage, device removal, or correction of anatomical abnormalities that perpetuate the infection [14,15,21,98,100,108,147,148,149].

Reassessment should determine whether persistent necrotic tissue, infected foreign material, drainable collections, anatomical leaks, vegetations, deep abscesses, or compartments with limited antifungal exposure are present. This assessment helps distinguish inadequate antifungal exposure from persistence of an anatomical focus. It may also prevent successive changes in antifungal therapy when the priority is surgical or interventional management or removal of infected material [15,21,46,63,100,108,109,148,149,150].

### 7.2. Early Surgical Intervention and Therapeutic Failure

Surgical and interventional procedures should be incorporated early into the therapeutic plan when the clinical condition, anatomical site, and technical feasibility support their use. The indication depends on the pathogen, the anatomical site and extent of the infection, the patient’s clinical stability, and the risk associated with the procedure. Early evaluation helps preserve the opportunity to achieve source control before progressive tissue involvement, increasing fungal burden, or clinical deterioration reduces the potential benefit of the intervention [15,63,66,67,149].

#### 7.2.1. Mucormycosis

Mucormycosis is a paradigmatic example of therapeutic failure associated with inadequate source control. Its angioinvasive nature causes thrombosis, ischemia, and necrosis, which reduce effective antifungal penetration into the affected tissue and promote local spread. For this reason, guidelines recommend the combination of active antifungal therapy, reversal of predisposing factors, and early and sufficiently extensive surgical debridement whenever feasible [15,63,66,149].

Recent regional recommendations identify the following pillars of management: early surgical debridement, induction therapy with liposomal amphotericin B (L-AmB), continuation therapy with ISA or POS where appropriate, and control of hyperglycemia and residual immunosuppression [15,63,67,150]. Delay in surgical intervention or inability to perform it can compromise the outcome, even when L-AmB therapy is initiated in a timely manner. In mucormycosis, persistent necrotic tissue acts as an infectious reservoir and limits the therapeutic response [15,66,149].

#### 7.2.2. Localized Invasive Aspergillosis

In IA, systemic antifungal therapy is the cornerstone of management; however, surgery may play a complementary role in selected situations, including focal pulmonary lesions at high risk of hemorrhage, localized disease that progresses despite appropriate treatment, bone or critical-structure involvement, and resectable residual lesions that may act as persistent foci [17,18,19,151].

In these scenarios, surgery can contribute to local control and reduce the risk of recurrence or anatomical complications. The decision to operate must be individualized based on immune status, the anatomical site and extent of the disease, the feasibility of complete resection, operative risk, and the availability of a multidisciplinary team experienced in managing complex IFIs [17,18,19,128,151].

### 7.3. Intravascular Devices and Biofilms

Biofilm formation on medical devices promotes persistent fungemia and an incomplete therapeutic response, particularly in candidemia and other forms of IC associated with catheters or intravascular materials. The organization of fungal cells within a biofilm reduces effective antifungal activity, hinders immune clearance, and allows the reservoir to persist despite adequate systemic antifungal exposure [14,21,96,97,98,146,152,153].

In candidemia, the decision to remove the CVC early must be individualized based on the patient’s clinical stability, the likelihood that the catheter is the source of infection, the availability of alternative access sites, and the risks associated with the procedure. When the CVC is considered the probable source—or when no other cause of persistent fungemia is identified—leaving it in place may delay microbiological clearance. Recent guidelines recommend removing the catheter as soon as this can be safely performed and recognize source control as an integral component of treatment [14,21,146,152,153,154].

Failure to remove an infected device or delays in its removal can result in microbiological persistence being misinterpreted as antifungal treatment failure. Changing or intensifying antifungal treatment without addressing the source can prolong fungemia and increase antifungal exposure without correcting the primary determinant of the poor treatment response [14,21,146,152,153,154].

### 7.4. Intra-Abdominal Candidiasis and Deep-Seated Collections

IAC frequently presents as a scenario of poor treatment response associated with insufficient source control. Persistent anastomotic leaks, perforations, abscesses, undrained collections, devitalized tissue, or infected surgical material can perpetuate the infection despite treatment with an active antifungal agent. The outcome depends on the interplay among timely initiation of treatment, adequate antifungal exposure within the abdominal compartment, and effective anatomical source control [100,101,148,150].

Changing the antifungal agent without addressing a persistent fluid collection or an active intra-abdominal source may fail to resolve the primary cause of the poor treatment response. Reassessment should include reviewing imaging studies, searching for leaks or residual collections, and assessing the need for percutaneous or surgical drainage when indicated and feasible. Coordination among clinical, surgical, and interventional radiology teams is essential to define the source control strategy [100,101,148,150].

IAC illustrates the overlap between pharmacological and anatomical determinants. Even when the causative organism is susceptible and the antifungal agent is active, local antifungal exposure may be insufficient in complex fluid collections, necrotic tissue, or cavities with inadequate drainage. Clinical progression must be interpreted in conjunction with imaging findings, surgical or interventional management, and microbiological results from clinically relevant samples [100,101,148,150]. Recent reviews of IAC in ICU patients highlight its diagnostic complexity, epidemiological shifts associated with non-albicans species, the importance of early treatment, and the PK/PD limitations of the peritoneal compartment. These factors support integrating anatomical source control and antifungal exposure when reassessing a poor treatment response [108,109].

### 7.5. Deep-Seated and Hard-to-Reach Foci

Deep-seated IFIs—such as hepatosplenic, renal, or intra-abdominal abscesses, osteoarticular infections, endocardial vegetations, and CNS lesions—pose challenges related to antifungal penetration, anatomical source control, and response assessment. At these anatomical sites, antifungal exposure may be variable, the local fungal burden may be high, and radiological resolution may be slower than clinical or microbiological improvement. Furthermore, some foci act as partially protected compartments in which systemic antifungal treatment alone may fail to achieve complete control of the infection. In selected patients with *Aspergillus*-related brain abscesses, neurosurgical drainage or resection should be considered when anatomically feasible and clinically indicated, particularly for accessible lesions associated with mass effect, diagnostic uncertainty, or an inadequate response to antifungal therapy [6,18,100,101,111,148,151,155].

When the focus is not amenable to drainage or is difficult to access, the therapeutic response may be gradual, and persistent imaging findings do not necessarily imply therapeutic failure. Therapeutic progress should be assessed by integrating clinical stability or improvement, serial microbiological and imaging findings, and the absence of new foci or signs of progression. Failure to recognize these limitations can lead to intensification of antifungal therapy or changes in the antifungal regimen that do not modify the primary anatomical determinant of the poor treatment response [6,18,100,101,111,148,155].

### 7.6. Endocarditis, Endophthalmitis, and Metastatic Foci

Some IFIs persist because of involvement of metastatic foci or structures that are difficult to clear, including heart valves, vascular endothelium, the retina, the vitreous, bone, and the CNS. In persistent or recurrent candidemia, endocarditis, endophthalmitis, septic thrombophlebitis, osteomyelitis, and deep-seated abscesses should be considered as part of a targeted search to distinguish inadequate antifungal exposure from persistent anatomical foci of infection [14,21,147,156,157].

Management depends on the anatomical site, extent of infection, and feasibility of an intervention and may include prolonging antifungal treatment, selecting an antifungal agent with adequate penetration, surgery, drainage, vitrectomy, or removal of infected prosthetic material. If these foci are not recognized in a timely manner, they can perpetuate the infection and cause a poor treatment response to be misinterpreted as primary antifungal failure [14,21,99,147,156,157].

The evaluation of persistent candidemia should not be limited to repeating blood cultures or modifying the antifungal treatment regimen. The search for metastatic foci should be guided by signs and symptoms, the duration of fungemia, the identified species, the presence of devices or prosthetic material, and the overall clinical evolution [14,21,147,156,157].

### 7.7. When Complete Control of the Focus Is Not Feasible

In some patients, complete source control is technically impossible or carries disproportionate risk. This may occur with extensive disease, involvement of critical structures, clinical instability, severe thrombocytopenia, multiorgan dysfunction, or the absence of a viable surgical option. Under these circumstances, persistent clinical, microbiological, or radiological abnormalities may reflect an anatomical or physiological limitation that cannot be corrected rather than modifiable treatment failure [6,15,100,108,150].

Recognizing these limitations allows therapeutic goals and treatment duration to be individualized and, when appropriate, supports consideration of salvage strategies, long-term disease control, or suppressive antifungal therapy. It also prevents repeated regimen changes when complete eradication is unattainable for anatomical or clinical reasons and supports realistic communication about prognosis, treatment goals, and the balance among expected benefit, toxicity, and treatment burden [6,15,24,63,67].

### 7.8. Multidisciplinary Approach and Timing of the Intervention

Source control requires coordination among infectious diseases, surgery, intensive care, interventional radiology, microbiology, and clinical pharmacy and, depending on the clinical scenario, ophthalmology, neurology, otolaryngology, hematology, or transplantation teams. Fragmented care may delay biopsy, drainage, resection, device removal, or treatment optimization, thereby contributing to persistence or progression of the IFI [6,24,26,28,146,148,153,154].

The timing of intervention is a central component of management. Delayed drainage, surgery, or device removal may reduce the benefit of an otherwise active antifungal agent. The feasibility of source control should be assessed at the beginning of treatment and reconsidered in patients with persistent fungemia, fever without improvement, clinical or radiological progression, or discordant biomarker trends [14,15,21,24,25,29,100].

In patients with an IFI and a suboptimal response, reassessment may identify retained devices, undrained collections, necrotic tissue, biofilms, or deep-seated foci requiring targeted intervention. Within an AFSP, this review helps determine whether the poor response is associated with a modifiable, partially modifiable, or non-modifiable anatomical limitation and may prevent unnecessary antifungal escalation. Table 2 summarizes the main domains associated with treatment failure and the priority actions for structured clinical reassessment.

## 8. Therapeutic Failure According to Clinical Entity

Although several determinants of antifungal treatment failure are shared across IFIs, their relative importance varies by clinical syndrome. Reassessment must therefore be adapted to the specific condition, as the predominant causes of a poor response differ among IA, persistent candidemia, CM, mucormycosis, infections caused by rare filamentous fungi, and disseminated histoplasmosis [4,5,6,8,14,15,17,18,19,20,21,25,158].

### 8.1. Rationale for a Clinical Entity-Based Approach

Antifungal treatment failure requires an analysis that is both overarching and tailored to the specific clinical syndrome. In IA, persistent immunosuppression, early diagnosis, adequate triazole exposure, and appropriate interpretation of imaging findings are particularly important; in persistent candidemia, source control and management of intravascular devices; in CM, fungal burden, induction therapy, and control of ICP; and in mucormycosis, early diagnosis, prompt surgical debridement, and correction of underlying metabolic abnormalities. In infections caused by rare filamentous fungi and in disseminated histoplasmosis, species-level identification, antifungal susceptibility, timely diagnosis, and host-related factors are particularly relevant [4,5,14,17,18,19,20,21,94,95,139,158,159,160].

This approach helps prioritize reassessment according to the determinants most likely to explain a suboptimal treatment response in each clinical syndrome. Rather than replacing disease-specific guidelines, it provides a structured framework for interpreting a poor treatment response before attributing it to antifungal resistance or an irreversible loss of treatment effectiveness [16,43].

### 8.2. Invasive Aspergillosis

In IA, a poor treatment response often results from the interplay of profound immunosuppression, diagnostic uncertainty, high fungal burden, inadequate triazole exposure, and difficulty interpreting imaging findings, particularly during the early stages of treatment or during immune recovery. Although antifungal resistance is a possible explanation, delayed diagnosis, lack of immune recovery, suboptimal antifungal exposure, and premature interpretation of imaging findings frequently play a more prominent role [17,18,19,23,32,59,62,74,107,128,131].

#### 8.2.1. Common Failure Patterns

Among the most frequent determinants of a poor treatment response are delayed or uncertain diagnosis, profound and persistent neutropenia, irreversible immunosuppression, high fungal burden, suboptimal triazole exposure, and—in certain epidemiological contexts—triazole resistance. Clinical and imaging responses may be slow, and an initial increase in lesion size does not necessarily imply disease progression, particularly during neutrophil recovery [18,19,32,39].

Reassessment must integrate the clinical course, serial imaging findings, GM testing where appropriate, antifungal exposure, TDM for triazoles, and immune status. Modifying antifungal treatment without reviewing these components may increase toxicity and delay the correction of modifiable factors, such as subtherapeutic antifungal exposure or persistent immunosuppression [18,19,59,62,161].

#### 8.2.2. Azole Resistance and Clinical Response

Triazole resistance in *A. fumigatus* should be considered in patients with previous azole exposure, breakthrough IFI, a poor treatment response, or epidemiological exposure in regions with a high prevalence of resistance. Its presence may contribute to treatment failure, but an unfavorable outcome may also be determined by advanced disease, high fungal burden, adverse host factors, or insufficient antifungal exposure [9,19,23,33,83].

When resistance is suspected, the evaluation may include cultures, species-level identification, antifungal susceptibility testing, and molecular assays to detect resistance mechanisms, depending on availability. These studies should complement a comprehensive clinical reassessment. Optimizing antifungal exposure through TDM and reducing immunosuppression, where feasible, remain relevant even while resistance is being investigated [9,19,23,31,33,74,83,161].

### 8.3. Invasive Candidiasis and Persistent Candidemia

In IC, particularly in persistent candidemia, a poor treatment response is often associated with insufficient source control, although inadequate antifungal exposure, antifungal resistance, and deep-seated or metastatic complications must also be considered. Persistent positive blood cultures necessitate a search for infected devices, endovascular foci, IAC, deep-seated abscesses, and other persistent anatomical sources [14,21,31,73,93,100,108,109,146,153,156,157].

#### 8.3.1. Persistence of Fungemia

Persistent candidemia is a manifestation of microbiological failure but does not necessarily imply antifungal resistance. It often reflects an uncontrolled source of infection, such as an infected CVC, endocarditis, septic thrombophlebitis, intra-abdominal infection, deep-seated abscesses, or infected prosthetic material. Reassessment must include a search for the source of infection, species-level identification, review of previous antifungal exposure, and antifungal susceptibility testing when indicated [14,100,108,153,155,156,157].

Recent global guidelines on candidiasis support an integrated management approach that includes microbiological confirmation, assessment of complications, source control, and early removal of the CVC when this can be performed safely [21]. The identified species may also have therapeutic implications; *N. glabratus* and *C. auris* can exhibit resistance or multidrug resistance, yet source control and appropriate antifungal exposure remain essential components of management [14,31,73,85,91,93,98].

#### 8.3.2. Reactive Therapeutic Changes

Prematurely changing the antifungal agent will not necessarily resolve persistent candidemia if an infected catheter, an intra-abdominal collection, endocarditis, or a deep-seated focus remains unaddressed. In such cases, escalating antifungal therapy may increase antifungal exposure and delay an intervention targeting the responsible source [14,45,100,109,146,155,157].

Reassessment must include repeated blood cultures, confirmation of species-level identification, review of antifungal susceptibility and exposure, a search for deep-seated or metastatic foci, and assessment of the need to remove infected devices. Any modification or intensification of antifungal treatment should be based on these findings and the clinical and microbiological context rather than solely on the persistence of fungemia [14,45,100,109,146,155,157].

### 8.4. Cryptococcal Meningitis

In CM, the outcome depends on the efficacy of induction therapy, the reduction of fungal burden in the CSF, the control of ICP, and the patient’s immune status. Clinical deterioration during treatment does not necessarily imply treatment failure or antifungal resistance; it requires differentiation among microbiological persistence, uncontrolled ICP, toxicity, neurological complications, and IRIS [20,34,69,75,162,163].

#### 8.4.1. Fungal Burden, Suboptimal Induction Therapy, and Slow Microbiological Clearance

A poor treatment response may be associated with a high fungal burden, slow microbiological clearance, or a suboptimal induction regimen. In many settings, the combination of L-AmB and 5-FC is the preferred induction regimen, and the absence of 5-FC, when indicated and available, may reduce the speed and magnitude of the microbiological response [20,69,75,76,164]. The global ECMM/ISHAM/ASM guideline identifies effective induction therapy, ICP control, and differentiation among microbiological persistence, relapse, and IRIS as core components of management [20].

Persistent symptoms do not automatically indicate antifungal treatment failure. Reassessment must consider microbiological viability in the CSF, ICP, toxicity, neurological complications, coinfections, and IRIS. Distinguishing among these factors helps avoid switching antifungal agents without addressing the primary driver of clinical deterioration [34,69,75,76,164].

#### 8.4.2. Intracranial Pressure and Neurological Deterioration

Control of ICP is a fundamental determinant of outcome in CM. Failure to perform therapeutic lumbar punctures, delays in their performance, late recognition of intracranial hypertension, or attribution of neurological deterioration solely to antifungal treatment failure can increase morbidity and compromise the clinical course, even when the induction regimen is appropriate [20,34,75,162,164].

In the event of neurological deterioration, reassessment must include measurement of opening pressure, therapeutic management of ICP, CSF cultures, fungal burden, immune status, and assessment for IRIS or other neurological complications. When uncontrolled ICP is the predominant mechanism, modifying the antifungal agent alone may not improve the clinical course [20,34,75,162,164].

### 8.5. Mucormycosis

The therapeutic response in mucormycosis depends heavily on timely diagnosis, anatomical extent, and the feasibility of achieving source control. Its angioinvasive nature, rapid progression, tissue necrosis, and association with diabetes, acidosis, neutropenia, or immunosuppression can limit the efficacy of antifungal treatment alone, particularly when diagnosis, initiation of antifungal therapy, or surgical intervention is delayed [15,63,66,67,149].

#### 8.5.1. Delayed Diagnosis and Angioinvasive Progression

Angioinvasion promotes thrombosis, ischemia, tissue necrosis, and rapid local progression. Even with an active antifungal agent, the therapeutic response may be limited when diagnosis is delayed, necrotic tissue persists, or treatment cannot be complemented by surgical management and correction of modifiable predisposing factors [15,63,66,67,103,149].

Delayed diagnosis also reduces the likelihood of performing an effective resection before the disease compromises critical structures. In rhino-orbito-cerebral, pulmonary, or extensive cutaneous forms, anatomical progression can be rapid, and necrosis limits antifungal penetration into the affected tissue. Early clinical suspicion and timely collection of samples for histopathology, culture, and molecular assays, when available, are essential to prevent damage that may become irreversible [15,63,66,67,103,149].

#### 8.5.2. Surgery, Metabolic Control, and Antifungal Treatment

Management requires an integrated strategy combining active antifungal treatment—typically L-AmB as initial therapy—early surgical debridement when indicated and feasible, and correction of modifiable predisposing factors. These factors include hyperglycemia, acidosis, iron overload, and potentially reversible immunosuppression. The absence or delay of any of these components can contribute to an unfavorable outcome [15,63,149,165].

In mucormycosis, treatment failure can rarely be attributed solely to the antifungal agent. The persistence of necrotic tissue, insufficient resection, delayed intervention, or inadequate metabolic control can limit the therapeutic response, even with an appropriate antifungal regimen [15,63,149,165].

### 8.6. Rare and Emerging Filamentous Fungi

IFIs caused by rare and emerging filamentous fungi are associated with a high risk of poor treatment response because of identification challenges, intrinsic resistance or unpredictable susceptibility profiles, limited clinical evidence, and the major influence of immune status. Reassessment must incorporate accurate species-level identification, antifungal susceptibility testing when indicated, source control, and specialist assessment before empirical changes to antifungal treatment are made [4,42,87,94,95,166]. Contemporary series from tertiary care centers show that these infections remain associated with high mortality and that *Fusarium* spp. and *Scedosporium* spp. are among the most clinically significant rare molds, particularly in patients with profound immunosuppression or disseminated disease [166].

#### 8.6.1. Fusariosis

Invasive fusariosis is associated with poor outcomes and high mortality, particularly in patients with profound neutropenia, hematologic malignancies, or hematopoietic stem cell transplantation. Determinants of a poor response include hematogenous dissemination, fungemia, metastatic skin lesions, variable antifungal susceptibility, and dependence on immune recovery. In many patients, prognosis depends more on reversal of neutropenia and other immune defects than on simply switching the antifungal agent [4,87,166].

Species-level identification and antifungal susceptibility testing can help guide treatment, although the correlation between in vitro results and clinical outcomes is not always direct. Combination therapy, regimen changes, and salvage strategies should be individualized according to disease extent, prior antifungal exposure, clinical response, and host-related factors, ideally with specialist input [4,87,166].

#### 8.6.2. Scedosporiosis and Lomentosporiosis

Infections caused by *Scedosporium* and *Lomentospora* are therapeutically challenging because of their variable susceptibility profiles and, in some species, intrinsic resistance to multiple antifungal agents. *Lomentospora prolificans* is associated with very limited treatment options and high mortality in disseminated disease. A poor response may therefore reflect both pathogen-related factors and the absence of therapeutic alternatives with predictable activity [4,87,94,166]. Recent reviews emphasize the importance of accurate species identification, assessment of disease extent, immune recovery, and source control. Antifungal agents currently under investigation may expand future therapeutic options, although their role still requires clinical confirmation [94].

Reassessment should confirm the species, review susceptibility results whenever available, determine the extent of infection, assess the need for surgery or drainage, and consider immune status. An empirical regimen change may be insufficient when the expected susceptibility profile is unknown or when other determinants of the poor response remain uncorrected [4,87,94,166].

#### 8.6.3. Other Rare Molds

Other rare or emerging filamentous fungi, including *Rasamsonia*, *Paecilomyces*, *Purpureocillium*, *Scopulariopsis*, and uncommon species within the order Mucorales, may cause IFIs characterized by difficult identification, limited therapeutic evidence, and variable susceptibility profiles. A poor response may result from delayed or incorrect identification, selection of an antifungal agent with insufficient activity, persistent immunosuppression, or the limited clinical evidence available to guide treatment [4,63,149,167,168,169].

When infection caused by a rare mold is suspected, evaluation should pursue precise identification, review antifungal susceptibility when appropriate, consider the organism-specific evidence available, and actively assess source control and host-related factors. These organisms should not be grouped into a single generic therapeutic category because they differ substantially in clinical behavior, antifungal susceptibility, and expected treatment response [4,63,149,167,168,169].

### 8.7. Disseminated Histoplasmosis and Other Endemic Mycoses

Disseminated endemic mycoses are particularly relevant in Latin America and in patients with advanced HIV or other forms of immunosuppression. A poor response may be associated with delayed diagnosis, high fungal burden, advanced disease, coinfections, insufficient azole absorption, limited access to specific diagnostic tests, or failure to use L-AmB induction therapy in severe disease [5,159,170,171,172].

#### 8.7.1. Disseminated Histoplasmosis

Disseminated histoplasmosis is particularly important in Latin America because of its association with advanced HIV and other immunosuppressive conditions and its frequent clinical overlap with TB, lymphoma, and other systemic diseases. Poor outcomes are often associated with delayed diagnosis, high fungal burden, advanced disease, coinfections, limited access to antigen detection, inappropriate use of ITC, or omission of L-AmB induction therapy in severe cases [5,135,136,139,160,171,173]. Recent reviews indicate that histoplasmosis in people with advanced HIV remains an important clinical and public health problem in the region, partly because of diagnostic delays, unequal access to antigen testing, and clinical overlap with TB and other opportunistic infections [136,138,173,174].

Detection of *Histoplasma* antigen in urine or serum can support earlier diagnosis and treatment initiation. When the response is unfavorable, reassessment should include adherence, ITC absorption and exposure, drug–drug interactions, initial disease severity, coinfections—particularly TB—and possible IRIS after ART initiation [5,135,136,139,160,171,173].

#### 8.7.2. Other Endemic Mycoses

Other endemic mycoses, including coccidioidomycosis, blastomycosis, paracoccidioidomycosis, talaromycosis, and emergomycosis, may also cause true or apparent treatment failure, particularly in disseminated disease, CNS involvement, advanced immunosuppression, or delayed diagnosis. Evaluation should integrate geographic exposure, clinical presentation, available diagnostic methods, disease severity, anatomical site, and selection of the antifungal regimen [5,136,159,172].

A poor response may be associated with an inadequate initial regimen, insufficient treatment duration, suboptimal azole absorption or exposure, involvement of anatomical sites with limited drug penetration, or persistent immunosuppression. When clinically indicated, TDM can help assess exposure to selected azoles, particularly in disseminated disease or infection involving complex anatomical sites [5,136,159,172].

### 8.8. Operational Summary by Clinical Entity

Reassessment must be tailored to each clinical entity. In IA, priorities include diagnostic certainty, immune status, radiological progression, and azole exposure. In persistent candidemia, priorities are the identification of uncontrolled foci, retained devices, and deep-seated or metastatic complications. In CM, it is essential to distinguish between microbiological persistence, uncontrolled ICP, and IRIS. In mucormycosis, priorities include anatomical extent, the timing of medical and surgical interventions, and correction of predisposing factors.

For infections caused by rare filamentous fungi, reassessment should emphasize species-level identification, susceptibility profiles, and immune status. In disseminated histoplasmosis, considerations include the timing of diagnosis, initial disease severity, access to specific diagnostic tests, treatment adequacy, and the possibility of coinfections or IRIS. Organizing reassessment by clinical syndrome complements rather than replaces a comprehensive assessment of the host, pathogen, antifungal exposure, and source control.

Table 3 summarizes the main patterns of poor response and reassessment priorities for each clinical entity.

## 9. Clinical Approach to a Suboptimal Response to Antifungal Treatment

When a suboptimal response is identified, any modification of the antifungal regimen must be based on a structured clinical reassessment. Persistent fever or fungemia, clinical deterioration, and radiological progression may reflect resistance, but may also arise from an incomplete diagnosis, insufficient antifungal exposure, persistent immunosuppression, high fungal burden, breakthrough IFI, or inadequate source control [6,15,17,18,21,22,35,46,63,109,115,121].

### 9.1. General Principles of Clinical Reassessment

When a patient does not respond to treatment considered appropriate, clinicians may consider switching antifungal agents, adding another agent, or prolonging therapy. However, these interventions do not necessarily address the underlying cause if the modifiable determinants of the poor treatment response have not first been identified. They may also increase toxicity, the risk of drug–drug interactions, costs, and selective pressure without providing a clear clinical benefit [6,10,13,24,25,47,49,101].

Reassessment must confirm diagnostic certainty, verify pathogen identification and its expected or documented susceptibility, reconstruct the antifungal exposure actually achieved, assess immune status, search for persistent foci, and reconsider alternative diagnoses or concomitant infections. Before treatment is intensified, all unconfirmed, suboptimal, or uncorrected aspects of the diagnostic and therapeutic process must be addressed [6,16,24,27,28,43].

These principles align with AFSPs, which promote targeted and timely prescribing, multidisciplinary collaboration, integration of diagnostic information, and systematic reassessment of the indication, antifungal exposure, and therapeutic response [24,26,27,28,29].

### 9.2. When Should a Lack of Response Be Considered to Exist?

A lack of response must be interpreted according to the pathogen, clinical syndrome, anatomical site, initial fungal burden, host-related factors, and expected time to improvement. Response kinetics vary among IFIs: persistent positive blood cultures in candidemia have different implications from early radiological progression in IA or neurological deterioration associated with elevated ICP in CM [14,15,18,19,20,21,32,34,39,63,77,162,163].

Formal reassessment is indicated when no improvement is observed after a reasonable period of otherwise appropriate treatment; when clinical or hemodynamic deterioration occurs; when fungemia or other evidence of viable infection persists; when new foci emerge; or when the clinical course is inconsistent with the initial diagnosis. Early deterioration requires immediate reassessment, whereas clinically stable patients may undergo a scheduled evaluation when appropriate. A period of 5–7 days without improvement may prompt reassessment in certain IFIs, but this is not a universal threshold and must be interpreted according to the syndrome, anatomical site, parameter being evaluated, and immune status [3,6,7,8,14,15,17,18,32,63,108,109,124].

Premature interpretation of the response can lead to unnecessary treatment modifications. In IA, an early increase in lesion size on imaging during neutrophil recovery may reflect inflammation rather than fungal progression. Similarly, persistent or fluctuating levels of antigens and other biomarkers do not, by themselves, demonstrate microbiological failure and must be interpreted according to biomarker kinetics, timing of measurement, antifungal exposure, and the overall clinical and radiological course [3,6,7,8,14,15,17,18,19,32,37,63,108,109,124,128,130].

### 9.3. Definition of the Objective and Timing of Reassessment

Once a suboptimal response is recognized, reassessment must have a specific objective and a defined timeframe for determining whether that objective has been achieved. The clinical team should specify the expected outcome—clinical stabilization, microbiological clearance, a favorable biomarker trend, source control, improvement or stabilization of imaging findings, or reversal of a host-related factor—and the interval at which it will be assessed. Defining these elements helps avoid prolonged interventions without documented benefit and distinguish a slow clinical course consistent with the syndrome from persistent treatment failure [3,6,8,9,24,25,28,30,36,37,103,129].

The objectives and timing of follow-up must be tailored to the clinical syndrome and the parameter being evaluated. In candidemia, these may include documented bloodstream clearance and source control; in CM, microbiological clearance and control of ICP; in mucormycosis, clinical stabilization, metabolic control, and surgery when indicated; and in IA, an integrated assessment of the clinical course, biomarkers, imaging findings, and immune recovery. Although the interval should be individualized, it should be defined explicitly at the start of the intervention [15,17,18,19,20,22,37,63,74,75,103,116,128].

The findings that would justify maintaining, optimizing, modifying, de-escalating, or discontinuing the antifungal strategy should also be predefined. These criteria help determine whether the intervention has addressed the main determinant of the poor treatment response and prevent prolonged treatment intensification without demonstrable clinical, microbiological, or radiological benefit [24,25,27,28].

### 9.4. Structured Reassessment Sequence

A structured reassessment of a suboptimal response can follow a reproducible clinical sequence. This sequence complements individual clinical assessment by organizing decision-making and identifying modifiable determinants before treatment is altered or intensified.

#### 9.4.1. Confirmation of the Diagnosis

Reassessment begins by determining whether the initial diagnosis remains valid. The clinical, radiological, microbiological, histopathological, and molecular criteria supporting the diagnosis of IFI must be reviewed, together with the quality and representativeness of the samples and the possibility that a positive result represents colonization or contamination. Alternative diagnoses and coinfections that could explain the clinical course must also be reconsidered [16,43,103,115,120,140,142].

When the clinical course differs from expectations, reassessment may require repeat cultures, broader sampling, collection of higher-yield specimens, higher-resolution identification methods, and the incorporation of biomarkers or molecular assays when appropriate. Concordance among the identified microorganism, clinical syndrome, and anatomical site must also be verified [16,103,115,120,121,124,125,126,127,140,141,142,145].

#### 9.4.2. Reassessing the Host

The next step is to assess the host’s immunological, physiological, and metabolic status. Persistent neutropenia, exposure to corticosteroids or other immunomodulators, transplantation, and active hematologic disease can limit the therapeutic response despite the use of an active antifungal agent [3,19,44,45,46,50,53,54,56,57]. Organ dysfunction, uncontrolled diabetes, and inability to reverse other predisposing factors can also compromise infection control and make it difficult to maintain effective and safe treatment [15,47,58,59,63,64].

Apparent failure associated with immune recovery must also be considered. During neutrophil recovery, reduction of immunosuppression, or initiation of ART, a paradoxical inflammatory response can mimic disease progression. Distinguishing this phenomenon from true fungal progression can prevent unnecessary changes in antifungal therapy and help direct treatment toward the predominant mechanism of clinical deterioration [34,68,69,70].

#### 9.4.3. Reassessing Antifungal Pharmacology

Before modifying the regimen, the antifungal exposure actually achieved must be assessed. This review should include the dose, route of administration, loading dose when applicable, adherence, absorption, renal and hepatic function, drug–drug interactions, toxicity, missed doses or treatment interruptions, changes in the route of administration, and continuity of access to therapy. In critically ill patients, transplant recipients, and patients with hematologic malignancies, standard dosing does not always ensure adequate antifungal exposure because pharmacokinetic variability may be substantial [10,11,47,49,59,60,61,62,104,113,114].

In critically ill patients, conventional dosing regimens can result in variable and, in some cases, subtherapeutic antifungal exposure. Review of antifungal pharmacotherapy in the ICU must consider dose individualization, changes in organ function, ECLS, drug–drug interactions, and TDM when indicated and available [47,59,60,61,62,112,113,114,175].

TDM is particularly relevant for VRC, POS, ITC, and 5-FC. However, TDM results must be integrated into the overall clinical assessment: a concentration within the therapeutic range does not compensate for an incorrect diagnosis, an uncontrolled infectious focus, a resistant pathogen, or an irreversible immune defect [48,59,60,62,107,161,175].

#### 9.4.4. Reassessing the Pathogen

A poor treatment response also requires reassessment of the causative microorganism. This reassessment should confirm species-level identification, consider cryptic or emerging species, evaluate intrinsic or acquired resistance, and include antifungal susceptibility testing when indicated and available. Previous antifungal exposure and the possibility of breakthrough IFI, mixed infection, or a pathogen outside the spectrum of activity of the current regimen must also be considered [73,82,83,86,103,122,124].

Pathogen reassessment is particularly important in persistent candidemia, aspergillosis with a suboptimal response to triazoles, infections caused by rare filamentous fungi, and episodes occurring during antifungal prophylaxis. The microorganism initially identified may not fully explain the clinical course, particularly when new foci, new clinical manifestations, or deterioration inconsistent with the initial diagnosis emerge [4,9,33,82,83,93,94,98,166].

#### 9.4.5. Reassessing Source Control

The need for source control must be assessed early and addressed promptly. Infected catheters or prosthetic material, undrained collections, deep abscesses, IAC, endocarditis, endophthalmitis, septic thrombophlebitis, osteomyelitis, or necrotic tissue can perpetuate infection despite the use of an active antifungal agent and adequate antifungal exposure [14,21,45,63,97,100,108,109,146,147,149,155,156,157,165].

Changing the antifungal agent without removing an infected device, draining a collection, or debriding necrotic tissue may fail to address the main determinant of the poor treatment response. Evaluation should include targeted imaging, investigation for deep-seated or metastatic foci, and early assessment by surgical or interventional radiology teams, as appropriate. Multidisciplinary discussion helps determine the feasibility, timing, and extent of the intervention [14,21,45,63,97,100,108,109,146,147,149,155,156,157,165].

### 9.5. Limited Role of Therapeutic Intensification

Intensification of antifungal therapy—whether by switching drug classes, adding another agent, or otherwise modifying the therapeutic strategy—should be considered only after systematic reassessment. In many cases, confirming the diagnosis, optimizing antifungal exposure, achieving source control, or reducing reversible immunosuppression may be more important than empirical treatment escalation [6,15,17,24,27,37,50,63,100,103,116,128,164].

If treatment is intensified, the clinical objective must be explicit: expanding coverage to include a pathogen outside the spectrum of the current regimen, treating documented or highly probable resistance, improving antifungal exposure at an anatomical site with limited drug penetration, addressing a breakthrough IFI, or using intensification as a temporary strategy while source control is being achieved. The timing of the next assessment must also be established, together with clinical, microbiological, and radiological criteria for maintaining, modifying, or de-escalating the intervention [22,31,63,73,74,82,100,108,109,165].

Combination antifungal therapy should be reserved for selected situations and individualized according to the pathogen, anatomical site, antifungal susceptibility, antifungal exposure, toxicity, drug–drug interactions, and available evidence. In the absence of a clear indication, combination therapy may increase toxicity, drug–drug interactions, and selective pressure without addressing the underlying cause of the poor treatment response [6,11,13,24,27,61,109,125].

De-escalation or discontinuation of antifungal treatment should be considered when reassessment rules out active IFI, identifies colonization or contamination, supports an alternative diagnosis, or fails to demonstrate benefit from treatment intensification. Within antifungal stewardship, appropriate narrowing of antifungal coverage and discontinuation of unjustified treatment are as important as timely initiation of therapy in patients with a high probability of IFI [24,25,26,27,28,29,30,176].

### 9.6. Clinical Checklist for Daily Practice

Checklists can prompt reassessment, reduce omissions, and facilitate coordinated decision-making among members of the multidisciplinary team. Although checklists do not replace clinical judgment, they help ensure that the key determinants of a poor treatment response have been reviewed before treatment is modified or intensified [24,25,26,27,28,29,30,176].

These tools should be used as clinical safety aids rather than rigid instruments. They can reduce anchoring bias, diagnostic omissions, and reactive treatment escalation while documenting the domains reviewed, determinants that remain uncorrected, interventions performed, and the scheduled time for reassessment.

Table 4 presents a practical checklist for the structured reassessment of patients with an IFI and a suboptimal response before antifungal treatment is maintained, modified, intensified, or discontinued.

## 10. Clinical Algorithms for the Evaluation of Antifungal Treatment Failure

In the absence of an explicit clinical sequence, an unfavorable clinical course can lead to empirical treatment changes, combination therapy without a defined rationale, unnecessary prolongation of therapy, or repeated diagnostic testing without a specific clinical question. Clinical algorithms help structure reassessment, prioritize potentially correctable factors, and align therapeutic decisions with the principles of AFSPs [24,25,26,27,28,29,30,176].

### 10.1. Rationale for an Algorithmic Approach

Antifungal treatment failure rarely stems from a single factor. Determinants related to the diagnosis, host, pathogen, antifungal exposure, and source control may coexist in the same patient. Treatment intensification alone may be insufficient unless accompanied by a systematic review of the diagnostic and therapeutic process [6,8,9,28,30,45,46].

Clinical algorithms do not replace individual clinical assessment or condition-specific recommendations. Instead, they structure decision-making, minimize omissions, and link each intervention to a clinical hypothesis, a therapeutic objective, and a defined time for reassessment. This structured approach supports decisions to maintain, optimize, intensify, de-escalate, or discontinue treatment [24,25,26,27,28,176].

Within AFSPs, these algorithms can identify recurring gaps, including diagnostic delays, failure to perform TDM when indicated, delays in source control, use of combination therapy without a specific rationale, and insufficient multidisciplinary coordination. They also help integrate diagnostic and pharmacological information and document the objectives that justify continuing or modifying treatment [24,25,26,27,28,29,30,59,60,62,107,161,175,176].

### 10.2. Cross-Cutting Algorithm for the Reassessment of Therapeutic Failure

The cross-cutting algorithm can be applied to any patient with IFI and a suboptimal response. It distinguishes true from apparent treatment failure, identifies potentially correctable determinants, and structures reassessment before treatment is maintained, optimized, modified, or discontinued. Although the algorithm is presented sequentially, several components must be addressed simultaneously when clinical severity requires urgent intervention [6,14,15,17,18,19,21,63].

The first step is to determine whether the observed clinical course represents a true lack of response. Interpretation must consider the time elapsed since treatment initiation, the clinical syndrome, initial fungal burden, immune status, and the expected kinetics of the clinical, microbiological, or imaging response [6,16,35,40,41]. In IA, early radiological progression may reflect neutrophil recovery rather than true fungal progression [18,19,32,39]. In CM, neurological deterioration may be associated with elevated ICP or IRIS; in persistent candidemia, failure to achieve microbiological clearance may indicate an uncontrolled intravascular or deep-seated focus [14,20,21,34,75,162,163,164].

When the clinical course is inconsistent with both an expected slow response and apparent treatment failure, the validity of the initial diagnosis must be reassessed. This review should consider the quality and representativeness of the specimens and the concordance among clinical, microbiological, radiological, and histopathological findings [16,36,103,115,119,123]. Colonization, contamination, mixed infection, and alternative diagnoses that could explain the clinical course must also be reconsidered [16,35,103,143,144,145]. If uncertainty persists, repeat sampling, collection of additional or higher-yield specimens, and higher-resolution identification or diagnostic methods may be required, depending on availability [74,86,120,121,126,140,141,142,145].

Once the diagnosis has been sufficiently established, host-related determinants of the therapeutic response must be assessed. Persistent neutropenia, drug-induced immunosuppression, uncontrolled diabetes, organ dysfunction, and inability to reverse predisposing factors may limit the therapeutic response despite the use of an active antifungal agent [3,15,46,50,53,54,63]. Conversely, immune recovery may trigger a paradoxical inflammatory response that mimics progression and must be distinguished from persistent viable infection [34,68,69,70].

In parallel, the pathogen must be reassessed. This includes confirming species-level identification and considering cryptic or emerging species [4,31,82,83,84,87,88,89,90,91,92]. The expected or documented antifungal susceptibility profile must also be reviewed, including intrinsic or acquired resistance [7,23,31,71,72,73,74,87,88,93]. Previous antifungal exposure, breakthrough IFI, mixed infection, or a microorganism outside the spectrum of the current regimen may provide an additional explanation for the poor treatment response [9,22,33].

The antifungal exposure actually achieved must also be assessed. This review should include dose, route of administration, loading dose when indicated, absorption, adherence, drug–drug interactions, toxicity, treatment interruptions, and renal and hepatic function [10,13,47,48,49,59,62,104,114]. A microbiologically active antifungal agent may still fail to achieve sufficient exposure in plasma or at the anatomical site because of pharmacokinetic variability or limited tissue penetration [49,99,100,101,110,111]. In critically ill patients, pathophysiological changes, renal replacement therapy, extracorporeal support, and TDM, when indicated and available, must also be considered [60,61,112,113,175].

The need for source control must be assessed concurrently. Infected devices, undrained collections, necrotic tissue, vegetations, prosthetic material, and deep-seated or metastatic foci can sustain infection despite adequate antifungal exposure [14,21,96,97,98,99,100,146,147,148,149,150,157]. When indicated, assessment by surgical or interventional radiology teams should occur early rather than after several empirical treatment changes [15,63,66,67,100,149,150].

**Figure 1:**
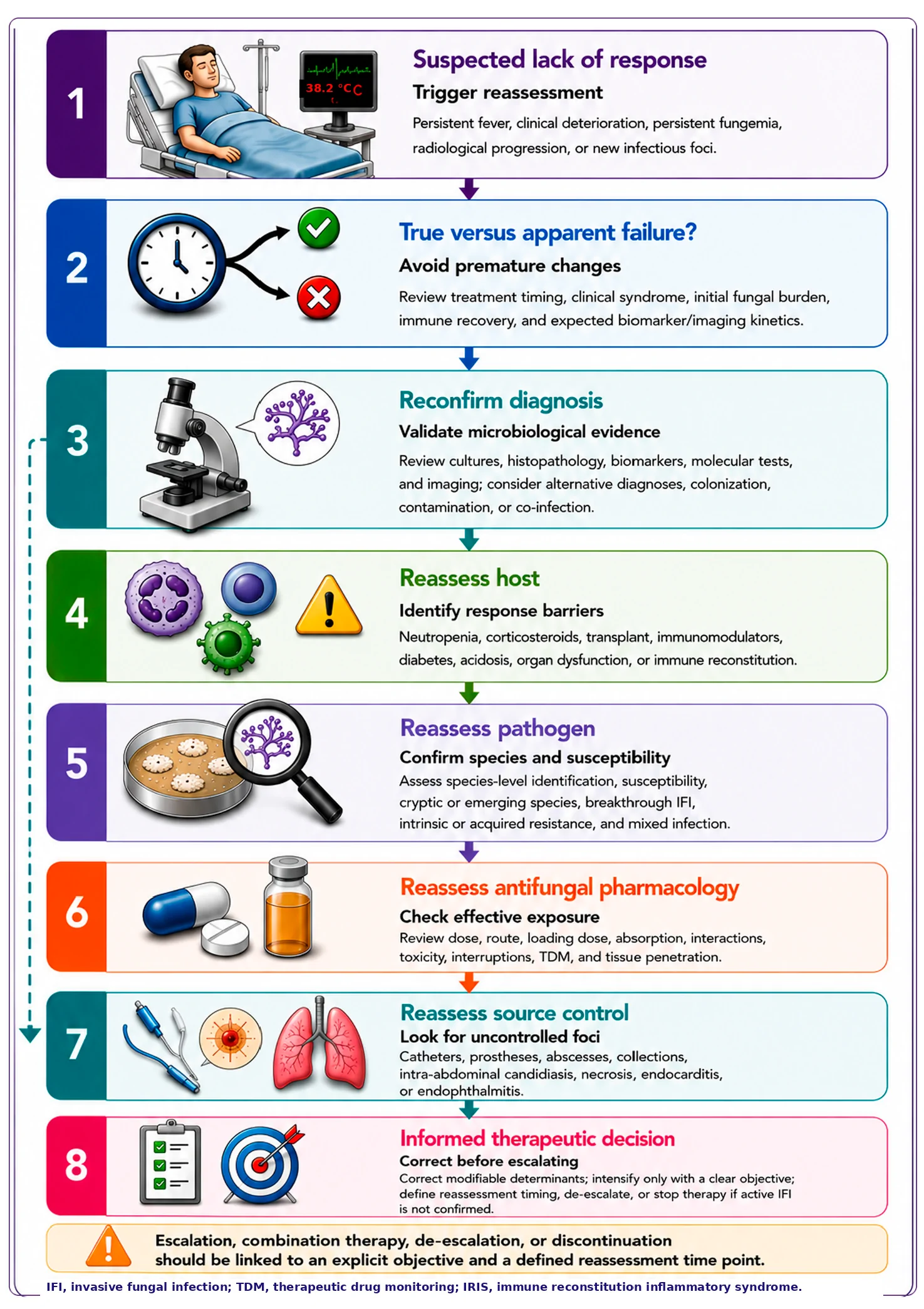
Cross-cutting algorithm for reassessing antifungal treatment failure. The algorithm presents a structured sequence for evaluating patients with invasive fungal infection and a suboptimal response to antifungal therapy. Reassessment begins by determining whether the observed clinical course represents true treatment failure rather than an expected slow response, paradoxical inflammation, early radiological changes, or transient biomarker persistence. Diagnostic certainty, host-related determinants, pathogen characteristics, antifungal exposure, and source control are subsequently reviewed, either sequentially or concurrently according to clinical severity. The final therapeutic decision should prioritize correction of modifiable determinants before antifungal treatment is intensified. Escalation, combination therapy, de-escalation, or discontinuation should be linked to an explicit clinical objective and a defined time for reassessment.

The final decision should address the predominant determinant of the poor treatment response. This may involve maintaining treatment when the clinical course is consistent with the expected response, optimizing antifungal exposure, broadening the diagnostic workup, or achieving source control. Modification of the regimen should be reserved for resistance, inadequate antifungal coverage, insufficient exposure, or confirmed disease progression [7,9,22,23,31,33,71,72,73,74]. When active IFI cannot be confirmed, colonization or contamination is identified, or an alternative diagnosis is established, de-escalation or discontinuation of antifungal treatment should be considered [24,25,26,27,28,29,30]. Each decision must be linked to an explicit clinical objective, predefined criteria for maintaining or modifying the intervention, and a defined time for reassessment [24,27,28,29,30]. The proposed cross-cutting algorithm summarizes this structured reassessment pathway and its main decision points (Figure 1).

### 10.3. Practical Application of the Cross-Cutting Algorithm

Application of the algorithm should be tailored to the patient’s clinical status and the syndrome being treated. When the clinical course does not follow the expected trajectory, it is necessary to determine whether the findings represent true treatment failure, apparent failure, or a slow response that remains consistent with the natural course of the disease. This structured reassessment reduces the likelihood of empirically switching the antifungal agent as the initial intervention and facilitates identification of potentially modifiable determinants [6,24,27,29,30,176].

In critically ill patients, the different components of reassessment should be evaluated concurrently. In persistent candidemia, repeat blood cultures, investigation for deep-seated foci, review of antifungal exposure, and assessment of the CVC can be undertaken in parallel. Similarly, in progressive mucormycosis, surgical intervention and correction of underlying metabolic abnormalities should be initiated promptly while diagnostic and microbiological characterization is being completed [9,14,15,21,33,63].

Each reassessment should result in a specific course of action: maintaining or optimizing the current regimen, adjusting antifungal exposure, expanding the diagnostic workup, achieving source control, modifying or intensifying treatment, or de-escalating or discontinuing antifungal therapy when active IFI is not confirmed. The decision, its intended objective, and the criteria for subsequent modification should be documented, thereby facilitating clinical review and audit within AFSPs [24,27,28,29,30,176].

### 10.4. Clinical Entity-Specific Algorithms

Although the overarching algorithm is applicable across IFIs, reassessment priorities vary according to the clinical syndrome. In IA, priorities include diagnostic certainty, clinical and radiological progression, immune status, and triazole exposure; in persistent candidemia, documenting bloodstream clearance, achieving source control, and removing intravascular devices when appropriate; in CM, distinguishing among microbiological persistence, uncontrolled ICP, and IRIS; and in mucormycosis, defining the anatomical extent of disease, ensuring timely antifungal treatment, performing surgery when indicated, and correcting predisposing factors. In infections caused by rare filamentous fungi and disseminated endemic mycoses, the same overarching framework applies, with particular emphasis on species-level identification, expected or documented antifungal susceptibility, epidemiological context, disease extent, and host-related factors [4,5,14,15,17,18,20,21].

Table S1 summarizes the reassessment priorities for these clinical entities, including common determinants of a poor response, the most relevant clinical pitfalls, and proposed corrective actions [4,5,42,95,136,138,159,160,170,177,178,179].

#### 10.4.1. Algorithmic Reassessment in Invasive Aspergillosis

In IA, reassessment must integrate diagnostic certainty, clinical progression, progression on imaging, immune status, and triazole exposure. Early radiological progression does not necessarily indicate treatment failure, particularly during neutrophil recovery, and must be interpreted together with the clinical course, serial imaging, GM testing where appropriate, and other microbiological findings [17,18,19,23,39,62,106,107].

The pharmacological review must consider the dose, route of administration, adherence, drug–drug interactions, and TDM of VRC or POS, where applicable. Neutropenia, exposure to corticosteroids or other immunosuppressive agents, inflammatory phenomena associated with immune reconstitution, and the epidemiological risk of azole resistance must also be reassessed [17,18,19,23,39,62,106,107].

If the clinical course remains unfavorable, alternative or concomitant diagnoses, breakthrough IFI, a pathogen outside the spectrum of the current regimen, insufficient antifungal exposure, and a localized focus amenable to intervention should be reconsidered. If resistance is suspected, cultures may be obtained and species-level identification, antifungal susceptibility testing, or molecular methods performed according to the clinical indication and their availability [23,72,82,120,124,142].

A change in antifungal class or the use of combination therapy should be linked to a defined clinical hypothesis and objective, such as addressing documented or highly probable resistance, correcting insufficient antifungal exposure, or treating a breakthrough infection. In selected cases involving localized disease or anatomical complications, surgical evaluation may be considered. This approach reduces the risk of modifying treatment solely because of progression on imaging or persistent biomarkers interpreted in isolation, particularly when the diagnosis remains probabilistic or azole exposure is uncertain [9,17,18,23,37,62,103,122,130].

#### 10.4.2. Algorithmic Reassessment in Invasive Candidiasis and Persistent Candidemia

In persistent candidemia, the management algorithm must prioritize identification and control of the infectious source. Persistently positive blood cultures require evaluation for a retained or infected CVC, septic thrombophlebitis, endocarditis, IAC, deep-seated abscesses, infected prosthetic material, and other metastatic foci, according to the clinical presentation. Simply changing the antifungal agent may fail to achieve microbiological clearance if the source remains uncontrolled [14,21,22,100,109,156,157]. Recent global guidelines on candidiasis support assessment for complications, documentation of microbiological clearance, and early removal of the CVC when it is considered a likely source and the procedure can be performed safely [21].

Reassessment must also confirm the species and review antifungal susceptibility where appropriate, particularly for species with a greater likelihood of resistance or multidrug resistance, such as *N. glabratus* and *C. auris*. The pharmacological review must include the dose, route of administration, renal and hepatic function, drug–drug interactions, achieved antifungal exposure, and the appropriateness of the antifungal agent for the anatomical site of infection [2,31,73,81,86,91,93].

In practical terms, the sequence involves repeating blood cultures until clearance is documented, confirming the species, reviewing antifungal susceptibility and previous exposure, assessing whether the CVC should be removed when it is a probable source, and investigating endovascular, intra-abdominal, osteoarticular, ocular, or prosthetic foci according to the clinical and microbiological findings. A change in antifungal therapy must be justified by an objective reason, such as documented or highly probable resistance, insufficient previous exposure, inadequate penetration at the anatomical site of infection, or confirmed disease progression. Treatment duration should be individualized according to microbiological clearance, source control, the anatomical site of infection, and the presence of deep-seated or metastatic complications [14,21,22,31].

#### 10.4.3. Algorithmic Reassessment in Cryptococcal Meningitis

In CM, clinical deterioration may be associated with persistent viable infection, suboptimal induction therapy, the absence of 5-FC when indicated, insufficient antifungal exposure, toxicity, elevated ICP, coinfections, or IRIS. Simply changing the antifungal agent without assessing ICP or microbiological evidence of viable infection in the CSF may fail to address the primary determinant of the unfavorable outcome [20,34,68,70,75,164].

Reassessment must distinguish among elevated ICP, microbiological persistence or relapse, toxicity, coinfection, and IRIS. Opening pressure should be measured and managed through therapeutic procedures when indicated. The composition and duration of induction therapy, use of 5-FC, adherence, drug–drug interactions, renal and hematological function, and immune status must also be reviewed [20,34,68,70,75,164].

CSF culture should be repeated when microbiological persistence or relapse is suspected, and the results must be interpreted in the context of the clinical course and treatment received. Persistent CrAg positivity does not, by itself, demonstrate viable infection and should not be used in isolation to define treatment failure. The timing of ART initiation, recent changes in immunosuppression, and the possibility of IRIS should also be reviewed before the antifungal strategy is modified [20,34,68,70,75,164].

Treatment modification must be based on microbiological persistence or relapse, inadequate induction therapy, insufficient antifungal exposure, suspected or documented resistance, dose-limiting toxicity, or inability to continue the regimen. This approach integrates antifungal optimization with ICP control and helps distinguish active infection from inflammatory complications associated with immune recovery [20,34,68,70,75,164].

#### 10.4.4. Algorithmic Reassessment in Mucormycosis

In mucormycosis, reassessment must begin early because diagnostic, therapeutic, or surgical delays may allow anatomical progression that is difficult to reverse. Assessment should focus on disease extent, persistent necrotic tissue, the adequacy of surgical source control, the need for additional procedures, and the reversibility of predisposing factors. Hyperglycemia, acidosis, clinically significant iron overload, and modifiable immunosuppression should also be corrected whenever possible [15,63,149,165].

Pharmacological intensification does not replace surgical debridement when surgery is feasible. A suboptimal response may be associated with persistent necrosis, incomplete resection, unrecognized disease extent, insufficient antifungal exposure, or persistent predisposing factors. Clinical decisions require early and repeated multidisciplinary assessment tailored to the location and severity of the disease [15,63,149,165].

The sequence involves confirming the diagnosis through histopathology, culture, and molecular methods when available; defining disease extent through targeted imaging; initiating or confirming treatment with an active antifungal agent—typically L-AmB for severe disease; and obtaining early surgical assessment, with additional interventions when necrotic tissue persists or resectable disease progresses. At the same time, metabolic abnormalities must be corrected and immunosuppression reduced whenever clinically feasible [15,63,149,165].

Changing antifungal agents, using salvage therapy, or employing combination therapy should complement—not replace—source control. These decisions must be based on a clear rationale, such as dose-limiting toxicity, confirmed disease progression, insufficient antifungal exposure, or inability to continue the initial regimen. When complete anatomical source control is not feasible, realistic therapeutic goals and a defined time for reassessment must be established. Recent regional guidelines continue to identify early diagnosis, L-AmB for severe disease, timely debridement, metabolic control, and reversal of predisposing factors as key pillars of management [67].

### 10.5. Integration with Antifungal Stewardship Programs

Cases involving a suboptimal response can serve as tracer events within AFSPs by prompting a multidisciplinary review of diagnostic quality, pathogen identification, antifungal exposure actually achieved, drug–drug interactions, use of TDM when appropriate, source control, and the rationale for any treatment modification or intensification [24,28,30,59,62,176].

Systematic analysis of these episodes can also reveal recurring gaps and guide the development of indicators adapted to each institution’s epidemiology, diagnostic capacity, and available resources. The implications of this approach for auditing, education, and care organization are discussed in the following section.

## 11. Clinical and Organizational Implications for Antifungal Stewardship Programs

The analysis of antifungal treatment failure should not be limited to determining whether the antifungal regimen must be changed. In clinical practice, a suboptimal response should prompt review of the entire diagnostic and therapeutic pathway, including clinical assessment, the timeliness and quality of diagnosis, pathogen identification, antifungal exposure, source control, multidisciplinary coordination, and therapeutic response monitoring. This perspective is particularly relevant to AFSPs, which seek not only to reduce inappropriate antifungal use but also to support safer diagnostic and therapeutic decisions for patients with IFI [24,25,26,27,28,29,30,62,176].

### 11.1. Reassessment of the Diagnostic-Therapeutic Process

In practice, an unfavorable outcome should not initially be attributed to insufficient antifungal activity. In many patients, a suboptimal response is explained by correctable or partially modifiable factors related to diagnosis, antifungal exposure, host-related factors, or source control [6,14,15,18,20,21].

Before proceeding with empirical escalation, antifungal combination therapy, or treatment prolongation, the clinical team should systematically review potentially modifiable factors. Depending on the context, this assessment may lead to expanding the diagnostic workup, optimizing antifungal exposure, requesting TDM, removing infected devices, controlling collections or necrotic tissue, and reducing immunosuppression when clinically feasible [14,15,21,24,32,39,46,62,100,109].

This approach is particularly relevant in persistent candidemia, mucormycosis, IA, and CM. In these conditions, a poor response may be driven, respectively, by an uncontrolled intravascular or deep-seated focus; insufficient debridement or persistent metabolic abnormalities; suboptimal triazole exposure or premature interpretation of imaging findings; and unrecognized or inadequately controlled elevated ICP. Changing the antifungal agent without addressing the predominant underlying factor may prolong an unfavorable course and increase toxicity without providing clear clinical benefit [14,15,21,34,68,149,164].

Operationally, reassessment must document the domain most likely to explain the poor response, the findings supporting that interpretation, the proposed intervention, and the expected outcome. This documentation helps distinguish justified intensification from reactive treatment modification and facilitates communication among the clinical, microbiological, pharmacological, surgical, and critical care teams involved in the patient’s care [24,28,176].

### 11.2. Role of Antifungal Stewardship Programs

AFSPs can transform the reassessment of treatment failure into a structured institutional practice. Their scope must extend beyond antifungal authorization and cost review to integrate mycological diagnostics, clinical pharmacology, infectious diseases, hospital pharmacy, intensive care, surgery, and infection control into a coordinated decision-making strategy [24,28,30,176].

When a suboptimal response occurs, the AFSP can facilitate multidisciplinary reassessment, reduce unwarranted variability, and promote de-escalation or discontinuation when active IFI is not confirmed, colonization is identified, or an alternative diagnosis is established. It can also improve documentation of complex decisions and prevent avoidable toxicity, clinically relevant drug–drug interactions, and unplanned treatment interruptions, particularly in patients with polypharmacy, organ dysfunction, or prolonged treatment courses [3,24,28,30,46,176].

Each episode of poor response can serve as a tracer event for reviewing the diagnostic and therapeutic pathway. The assessment must determine whether the diagnosis and pathogen were confirmed, whether antifungal exposure was adequate, whether drug–drug interactions or toxicity occurred, whether source control was achieved, and whether host-related factors could be modified. When treatment is intensified, the rationale, expected objective, timing of reassessment, and criteria for maintaining, modifying, or de-escalating the intervention must be documented [6,24,28,30,176].

This approach expands the traditional role of AFSPs and establishes them as decision-support mechanisms in situations of substantial uncertainty. AFSPs contribute by integrating microbiological, clinical, and pharmacological information and linking a poor response to a specific, evaluable intervention [24,28,30,176].

### 11.3. Quality of Care Indicators Related to Treatment Failure

Systematic analysis of treatment failure can guide the development of quality indicators for the care of patients with IFIs. These measures should not be limited to antifungal prescribing but should encompass the timeliness and quality of the entire diagnostic and therapeutic process. Cross-cutting components may include the time required to obtain adequate specimens and initiate active antifungal therapy, species-level identification, performance and interpretation of antifungal susceptibility testing when appropriate, appropriate use of TDM, and time to source control [3,24,28,30,46,62,124,125,176].

Disease-specific measures may also be established according to the clinical syndrome. For candidemia, these measures may include follow-up blood cultures, catheter assessment, evaluation for deep-seated foci, and documentation of microbiological clearance; for IA, timely access to GM testing, thoracic imaging, and TDM for selected azoles; for mucormycosis, tissue sampling, initiation of L-AmB, surgical assessment, and metabolic control; and for CM, measurement and control of ICP, appropriate induction therapy, and microbiological monitoring where applicable [15,16,20,21,32,36,37,59,62,103,120,129,142].

Monitoring these measures can facilitate early identification of gaps in care instead of restricting evaluation to episodes in which clinical deterioration has already occurred. This approach is consistent with diagnostic stewardship principles, which integrate diagnostic quality and timeliness with the rational use of antifungals [3,24,30,37,46]. Table S2 presents potential indicators for AFSPs in treatment failure scenarios; these indicators should be interpreted as adaptable tools rather than universal standards or rigid thresholds [14,15,16,17,18,21,24,30,35,43,49,62,100]. Their implementation must consider local epidemiology, diagnostic capacity, institutional complexity, and available resources.

In addition to measuring compliance, these indicators should facilitate clinical feedback and continuous improvement. Their primary utility lies in identifying the stage at which a missed opportunity occurred—ranging from clinical suspicion and specimen collection to identification, antifungal susceptibility testing, TDM, surgical source control, or device removal. This information can guide educational interventions, changes in care processes, and specific institutional actions [24,28,62,124,176].

### 11.4. Educational and Organizational Implications

Preventing and promptly addressing antifungal treatment failure requires educational strategies for professionals involved in the care of patients with IFIs. These activities must strengthen early clinical suspicion, selection and collection of appropriate specimens, critical interpretation of biomarkers, recognition of emerging species and species with clinically relevant resistance profiles, application of pharmacokinetic and pharmacodynamic principles, and identification of foci requiring intervention [3,24,25,30,46].

Training should not be limited to disseminating therapeutic recommendations. It must also promote sequential diagnostic reasoning, dynamic interpretation of the therapeutic response, appropriate use of TDM, recognition of apparent failure, and formulation of explicit therapeutic goals. This approach can reduce empirical escalation, combination therapy without a clear indication, and prolongation of treatment without documented benefit [3,24,25,26,30,46].

A key educational component is distinguishing among a true suboptimal response, a slow response consistent with the expected clinical course, and apparent failure. This distinction can prevent premature treatment changes based on isolated persistent fever, positive biomarkers, or early radiological progression that is inconsistent with the overall clinical course [6,16,18,19,32,35,36,37,39,128,130]. It also supports contextual interpretation in patients with profound immunosuppression, immune recovery, or infection involving complex anatomical sites [3,34,44,45,46,68,69,70,100,101].

From an organizational perspective, institutions must establish pathways that ensure timely access to the resources required at their level of complexity. These resources include specialized microbiology, rapid identification, antifungal susceptibility testing, biomarkers, molecular methods, TDM, imaging, interventional radiology, and surgical support. When any of these capabilities are unavailable locally, mechanisms for referral, specimen transport, or specialized consultation must be established. The usefulness of clinical algorithms depends on obtaining the necessary diagnostic information and interventions within a clinically relevant timeframe [3,24,25,30,46].

Integration of AFSPs with infection control committees, the microbiology laboratory, clinical pharmacy, intensive care, surgery, and institutional quality programs can facilitate periodic review of cases involving a suboptimal response. Such reviews can identify recurring patterns, including diagnostic delays, inappropriate use of TDM, delays in source control, and inadequate documentation of therapeutic goals. The findings can guide educational activities, clinical audits, and targeted modifications to care processes [3,24,25,26,27,28,29,30,46].

### 11.5. Implementation Barriers and Adaptation to the Local Context

Implementing a structured approach to antifungal treatment failure depends on each institution’s diagnostic, pharmacological, and organizational capabilities. In resource-limited settings, access to biomarkers, molecular methods, rapid identification, antifungal susceptibility testing, TDM, broad-spectrum antifungals, interventional radiology, and specialized surgery may vary. AFSPs must therefore prioritize feasible, high-impact interventions, such as strengthening early clinical suspicion, improving specimen collection and processing, facilitating communication with the microbiology laboratory, and reviewing drug–drug interactions [3,24,25,26,27,28,29,30,46,59,62,104,124,125].

This adaptation does not entail accepting a lower standard of care but rather organizing available resources, defining clinical priorities, and establishing realistic referral pathways. A structured reassessment helps determine which decisions can be managed locally, which tests should be prioritized, which specimens require shipment to reference laboratories, and when specialist support or transfer to a higher-complexity center is necessary. This approach is particularly relevant in regions with a high burden of endemic mycoses, unequal access to advanced mycological diagnostics, and limited availability of antifungal agents or TDM [3,24,25,26,27,28,29,30,46,59,62,104,124,125].

Gaps in access to diagnostic tests and antifungal treatment are not confined to lower-complexity institutions. Recent reports from the World Health Organization have highlighted the need to expand access to diagnostic tools and therapies for IFIs. This situation underscores the importance of strengthening referral networks, specimen referral mechanisms, continuity of medication supplies, and regional diagnostic capacity while adapting AFSPs to the specific conditions of each center [2,180].

Evaluation of these strategies and their clinical, organizational, and care-delivery impact constitutes a research priority, as detailed in the following section.

## 12. Knowledge Gaps and Research Priorities

Important clinical and methodological gaps persist. Collectively, they hinder the definition, early recognition, and management of antifungal treatment failure. A key limitation is the absence of standardized definitions applicable across different clinical syndromes to distinguish among primary, secondary, and apparent failure, breakthrough IFI, and other forms of suboptimal response. This heterogeneity limits comparisons across studies, complicates outcome interpretation, and prevents the design of interventions that are reproducible across populations and centers [6,24,30].

Prospective evidence regarding structured reassessment strategies remains limited. Although guidelines and consensus statements support comprehensive assessment of diagnostic certainty, the host, the pathogen, antifungal exposure, and source control, it remains unclear whether systematic use of checklists, clinical pathways, or multidisciplinary models improves survival, clinical or microbiological response, safety, antifungal use, or hospital costs [17,18,20,21].

Another priority is to define how biomarkers, molecular methods, and rapid identification techniques should be integrated into clinically applicable algorithms. In practice, these tools are often used in a fragmented manner, without uniform criteria for interpreting trends, discordant results, persistence during treatment, or potential false-positive and false-negative results in the context of a suboptimal clinical response [16,20,24,36,37,103,140,141,142,174].

The clinical performance of these tests depends not only on their analytical characteristics but also on the timing of test ordering, the quality and representativeness of the specimen, prior antifungal exposure, the presence of a clearly defined clinical question, and timely communication between the laboratory and the treating team. Future studies should evaluate diagnostic stewardship strategies that integrate conventional and rapid methods with explicit criteria for initiating, maintaining, modifying, de-escalating, or discontinuing treatment, particularly in situations of high diagnostic uncertainty [24,28,30,103,176].

The clinical role and impact of TDM also require clearer definition. Although TDM is recommended for certain azoles and for 5-FC in selected settings, questions remain regarding its optimal implementation, the timing of measurements, exposure targets according to clinical syndrome and anatomical site, interpretation of results in critically ill patients, and its effect on clinical outcomes. These questions are particularly relevant in healthcare systems with limited access to TDM [59,62,104].

In critically ill patients, transplant recipients, and individuals with hematologic malignancies, future studies must determine how to individualize antifungal exposure according to the clinical syndrome, site of infection, changes in organ function, ECLS, drug–drug interactions, and cumulative toxicity. Pharmacokinetic studies conducted in the ICU show that conventional doses can result in variable exposures, supporting the need for pragmatic trials to evaluate whether strategies based on TDM, PK/PD targets, and early dose adjustment affect clinically relevant outcomes [49,61,101,112].

It is also necessary to standardize and validate measures of quality of care related to treatment failure. Priority domains include the timeliness of diagnosis and treatment, pathogen identification and antifungal susceptibility assessment, appropriate use of TDM, source control, and documentation of the rationale and objectives for treatment intensification. Before these measures are used as performance indicators or for institutional comparisons, they must demonstrate validity, feasibility, and the ability to reflect clinically relevant differences [24,46,49,101,108,109].

Research must also determine how these strategies should be adapted to institutions with differing diagnostic and therapeutic capabilities. Disparities in access to mycological and molecular testing, active antifungal agents, TDM, specialized surgery, and referral networks can affect diagnostic timeliness and the likelihood of a favorable response. Recent World Health Organization reports on priority fungal pathogens, diagnostic tests, and antifungal medicines underscore that these limitations represent a global challenge and must be incorporated into research, implementation, and public health agendas [2,180].

Addressing these gaps will require collaborative multicenter studies, pragmatic designs, and consistently defined outcomes. Priorities should not be limited to the development of new antifungal agents but must also include interventions that optimize diagnosis, antifungal exposure, source control, integration of mycological diagnostic methods, and access to specialized care through models adapted to local epidemiology and available resources.

## 13. Conclusions

Antifungal treatment failure is rarely explained solely by resistance. More often, it results from the interplay of delayed or incomplete diagnosis, host-related factors, pathogen characteristics, insufficient antifungal exposure, and inadequate source control. Toxicity, treatment interruptions, and limited penetration into the site of infection may also contribute. Therefore, an unfavorable clinical course should prompt a comprehensive review of the diagnostic and therapeutic process rather than automatically leading to antifungal escalation, combination therapy, or substitution.

Distinguishing among primary, secondary, and apparent failure, breakthrough IFI, and an expected slow response allows a more accurate interpretation of the clinical course and reduces unjustified treatment modifications. When a suboptimal response occurs, reassessment should confirm the diagnosis, characterize the pathogen, reconstruct the antifungal exposure achieved, assess the host’s capacity to respond, and determine whether source control has been achieved.

Clinical algorithms, checklists, and quality indicators can support more structured and well-documented decision-making and facilitate de-escalation or treatment discontinuation when active IFI is not confirmed. These tools can also improve multidisciplinary coordination and support the integration of treatment-failure reassessment into AFSPs. Their implementation must be tailored to local epidemiology, diagnostic and therapeutic capabilities, and the specific care gaps of each setting.

Within this framework, a suboptimal response serves as the starting point for identifying modifiable determinants and directing specific interventions. A systematic, multidisciplinary reassessment linked to explicit goals can improve both individual patient care and institutional processes for managing IFIs.

## Figures and Tables

**Table 1:** Conceptual Framework and Operational Classification of Antifungal Treatment Failure in Invasive Fungal Infections [6,14,16,18,19,21,22,32,35,43].

Domain	Operational Definition	Key Clinical Features	Implications for Reassessment
Clinical failure	Persistence or progression of signs and symptoms attributable to the IFI despite antifungal treatment considered appropriate for the clinical context.	Persistent fever, respiratory deterioration, hemodynamic instability, neurological involvement, or development of new sites of infection.	Do not initially assume antifungal resistance; reassess diagnostic certainty, host-related factors, antifungal exposure, and source control.
Microbiological failure	Persistent fungal isolation, ongoing fungemia, or failure to clear viable organisms from a clinically relevant specimen.	Persistently positive blood cultures or follow-up cultures; repeated recovery of the same pathogen from an appropriate specimen. Persistent biomarkers are complementary findings and do not, by themselves, demonstrate viable infection.	Search for a deep-seated focus or infected device; confirm the species; review antifungal susceptibility when appropriate; and verify antifungal exposure.
Radiological failure	Lack of improvement or progression of lesions on follow-up imaging.	Development of new lesions, increasing infiltrates, progression of nodules, cavitation, or local extension of disease.	Interpret findings according to treatment duration, clinical syndrome, baseline disease burden, and immune status; avoid defining failure solely on the basis of early imaging findings.
Primary failure	Absence of the expected clinical, microbiological, or radiological response during the initial period after starting treatment considered appropriate.	Persistent clinical instability, failure of microbiological clearance, or unfavorable radiological evolution, interpreted according to the clinical syndrome.	Reconfirm the diagnosis; review the antifungal agent, dose, loading dose, route, drug–drug interactions, TDM when appropriate, and source control.
Secondary failure	Loss of an initially favorable response, followed by clinical, microbiological, or radiological relapse or progression.	Recurrence of fever, recurrent fungemia, or relapse at a protected or deep anatomical site after an initial response.	Search for a persistent focus or anatomical relapse; review treatment interruptions, adherence, pharmacokinetic changes, acquired resistance, and the possibility of a breakthrough IFI.
Breakthrough IFI	A new IFI that develops during exposure to an antifungal administered for prophylaxis or treatment.	New IFI during exposure to a triazole, an echinocandin, or L-AmB; infection caused by an off-spectrum or resistant pathogen; or infection favored by insufficient antifungal exposure.	Do not automatically classify the episode as resistance; review antifungal spectrum and exposure, TDM when appropriate, adherence, drug–drug interactions, species identification, and antifungal susceptibility.
Apparent failure	Clinical, microbiological, or radiological evolution consistent with expected response kinetics but misinterpreted as a poor response.	Early persistent fever, early radiological worsening in aspergillosis, residual biomarker positivity, or inflammation associated with immune recovery.	Avoid premature treatment changes; integrate the time course, clinical syndrome, biomarker trends, imaging findings, baseline fungal burden, and immune status.

Note: Persistence or isolated variation of biomarkers does not, by itself, demonstrate viable fungal infection or microbiological failure and should be interpreted according to the clinical syndrome, timing of measurement, antifungal exposure, and the patient’s overall course. IFI, invasive fungal infection; L-AmB, liposomal amphotericin B; TDM, therapeutic drug monitoring.

**Table 2:** Main Determinants of Antifungal Treatment Failure: A Multidimensional Approach [4,6,14,15,16,18,19,23,24,30,31,49,62,91,98,99,100,101,103,143].

Domain	Clinically Relevant Examples	Predominant Mechanism	Priority Action for Reassessment
Host	Profound or persistent immunosuppression; critical illness; diabetes or metabolic acidosis; renal or hepatic dysfunction.	Inability to contain fungal invasion; altered inflammatory response; tissue progression; toxicity or inability to sustain treatment; absent, insufficient, or paradoxical immune recovery.	Determine whether the host-related defect is reversible, partially reversible, or non-modifiable; correct modifiable factors and adapt treatment according to organ function, toxicity, immune status, and prognosis.
Pathogen	High fungal burden; cryptic or emerging species; rare molds; intrinsic or acquired resistance; breakthrough IFI; biofilm formation.	Persistent infection or slow microbiological clearance; reduced susceptibility; infection caused by an organism outside the antifungal spectrum; incomplete, inaccurate, or discordant identification.	Confirm species-level identification whenever feasible; review antifungal susceptibility, previous antifungal exposure, and spectrum of activity; and interpret the findings according to fungal burden, anatomical site, epidemiology, and clinical syndrome.
Antifungal pharmacology	Insufficient dose or loading dose; poor absorption; drug–drug interactions; PK/PD variability; absent or delayed TDM; limited tissue penetration; toxicity; treatment interruptions.	Subtherapeutic systemic or tissue exposure; mismatch between antifungal distribution and the infected compartment; dose-limiting toxicity; incomplete or unsustained exposure.	Verify the antifungal agent, dose, loading dose, route, adherence, absorption, drug–drug interactions, organ function, toxicity, and treatment interruptions; request TDM when appropriate; and reconsider the agent or formulation according to the anatomical site and expected tissue penetration.
Diagnosis	Delayed clinical suspicion; inadequate or non-representative specimen; misinterpretation of a negative culture; incomplete or inaccurate pathogen identification; isolated interpretation of a biomarker result; syndromic misclassification.	Delayed or inappropriate treatment; incorrect antifungal selection; false perception of treatment failure; delayed source control; unnecessary treatment escalation.	Reconfirm the diagnosis; repeat or expand specimen collection; and integrate microbiology, histopathology, species identification, biomarkers, molecular methods, imaging, antifungal exposure, and the overall clinical context.
Source control	Infected device or prosthesis; biofilm; undrained collection; intra-abdominal candidiasis; necrotic tissue; endocarditis; endophthalmitis; osteomyelitis.	Anatomical persistence despite an active antifungal agent; limited penetration into necrotic tissue, vegetations, collections, protected compartments, or biofilms.	Prioritize device removal, drainage, debridement, or surgery when feasible; actively search for metastatic or deep-seated foci; and coordinate multidisciplinary assessment.
Healthcare system and care process	Delayed clinical suspicion; fragmented care; absence of a diagnostic pathway; limited access to diagnostic tests or TDM; delays in surgery or interventional procedures; unstructured reassessment.	Delayed clinical decisions; inefficient use of resources; avoidable toxicity; unnecessary selective pressure; persistence of correctable diagnostic, pharmacological, or anatomical determinants.	Activate multidisciplinary reassessment; use a structured checklist; and integrate diagnostic review, pharmacological optimization, source control, documentation, audit, and feedback within the AFSP.

Note: The determinants described above may coexist and may differ in their relative importance across patients and clinical entities. Priority actions should be individualized according to disease severity, the reversibility of identified factors, prognosis, and available resources. AFSP, antifungal stewardship program; IFI, invasive fungal infection; PK/PD, pharmacokinetics/pharmacodynamics; TDM, therapeutic drug monitoring.

**Table 3:** Main Patterns of Antifungal Treatment Failure According to Clinical Entity [4,5,6,14,15,17,18,20,21,23,31,32,37,62,87,91,100,136,166,168].

Clinical Entity	Main Determinants of Poor Response	Common Clinical Error	Reassessment Priority
Invasive aspergillosis	Delayed or uncertain diagnosis; persistent immunosuppression; suboptimal triazole exposure; antifungal resistance in specific settings; complex interpretation of imaging findings.	Interpreting early radiological progression as true treatment failure without considering neutrophil recovery, baseline disease burden, antifungal exposure, or biomarker trends.	Reconfirm the diagnosis; integrate imaging findings, GM trends, immune status, and the overall clinical course; optimize antifungal exposure through TDM when appropriate; and assess antifungal susceptibility in patients with a poor response or relevant epidemiological risk.
Invasive candidiasis and persistent candidemia	Retained CVC; deep-seated focus; intra-abdominal candidiasis; endocarditis; thrombophlebitis; species with reduced antifungal susceptibility or multidrug resistance.	Changing or escalating antifungal treatment without repeating blood cultures, searching for and controlling the infectious focus, or assessing the need for device removal.	Document bloodstream clearance; confirm species identification and antifungal susceptibility when appropriate; search for deep-seated or metastatic foci; achieve source control; and ensure adequate antifungal exposure.
Cryptococcal meningitis	High initial fungal burden; slow CSF clearance; suboptimal induction therapy; absence of 5-FC when indicated; elevated ICP; IRIS.	Attributing neurological deterioration solely to antifungal treatment failure without measuring and controlling ICP or distinguishing microbiological persistence from paradoxical inflammation.	Optimize induction therapy; measure and control ICP; reassess CSF findings when clinically appropriate; and distinguish among microbiological persistence, relapse, treatment toxicity, uncontrolled ICP, and IRIS.
Mucormycosis	Delayed diagnosis; angioinvasion; tissue necrosis; absent or delayed surgery; diabetes; acidosis; iron overload; or persistent immunosuppression.	Relying on pharmacological treatment alone without timely surgical debridement or correction of predisposing factors.	Initiate an active antifungal agent promptly; define the anatomical extent of disease; perform surgical debridement when feasible; and correct modifiable metabolic or immunological factors.

Note: Reassessment priorities should be adapted to disease severity, anatomical site, immune status, initial fungal burden, antifungal exposure, expected response kinetics, and the feasibility of source control. 5-FC, flucytosine; CSF, cerebrospinal fluid; CVC, central venous catheter; GM, galactomannan; ICP, intracranial pressure; IRIS, immune reconstitution inflammatory syndrome; TDM, therapeutic drug monitoring.

**Table 4:** Practical Checklist for the Structured Reassessment of Antifungal Treatment Failure [6,14,15,18,19,21,22,24,28,30,49,62,100].

Reassessment Domain	Key Clinical Question	Suggested Action
Diagnostic confirmation	Does the initial diagnosis of IFI remain valid?	Review the clinical, radiological, microbiological, histopathological, and molecular criteria; consider alternative diagnoses, coinfections, colonization, or contamination.
Pathogen	Was the causative fungus identified with sufficient precision?	Confirm species-level identification whenever feasible; review antifungal susceptibility when appropriate; and consider cryptic or emerging species, rare molds, mixed infection, or breakthrough IFI.
Host	Does the patient’s immunological and physiological status permit an adequate response?	Reassess neutropenia, corticosteroid exposure, immunomodulatory therapies, transplantation status, diabetes, acidosis, organ dysfunction, and the reversibility of the host-related defect.
Antifungal pharmacology	Was antifungal exposure adequate and sustained?	Verify the antifungal agent, dose, loading dose, route, absorption, adherence, drug–drug interactions, toxicity, treatment interruptions, route changes, renal and hepatic function, and the need for TDM.
Anatomical site and penetration	Is adequate antifungal exposure expected at the site of infection?	Review expected penetration into the CNS, eye, bone, endocardium, abscesses, abdominal cavity, necrotic tissue, and biofilms.
Source control	Is there a persistent or uncontrolled infectious focus?	Search for infected devices or prostheses, undrained collections, intra-abdominal candidiasis, endocarditis, endophthalmitis, thrombophlebitis, osteomyelitis, or necrotic tissue; assess the need for drainage, device removal, debridement, or surgery.
Apparent failure	Could the clinical course reflect an expected slow response or paradoxical inflammation rather than true treatment failure?	Interpret fever, biomarkers, and imaging findings according to the clinical syndrome, treatment duration, baseline fungal burden, immune recovery, and overall host status.
Care process	Is reassessment coordinated, multidisciplinary, and adequately documented?	Promote multidisciplinary discussion involving infectious diseases, microbiology, clinical pharmacy, intensive care, surgery, interventional radiology, and the AFSP.
Treatment intensification	Is there an objective reason to change, combine, or broaden antifungal treatment?	Define the indication, therapeutic objective, expected duration or exit criterion, reassessment time point, and de-escalation plan.
De-escalation or discontinuation	Does reassessment exclude active IFI or identify a more likely alternative explanation?	De-escalate, adjust, or discontinue treatment when the available evidence does not support active IFI or when treatment intensification is not expected to provide clinical benefit.
Follow-up plan	When will the response be reassessed, and which criteria will guide the next decision?	Document the timing of the next reassessment and the clinical, microbiological, radiological, and pharmacological criteria for maintaining, modifying, de-escalating, or discontinuing treatment.

Note: Several domains may require simultaneous assessment in patients with severe or rapidly progressive disease. Completion of the checklist should not delay urgent antifungal treatment, source control, or other time-sensitive interventions. AFSP, antifungal stewardship program; CNS, central nervous system; IFI, invasive fungal infection; TDM, therapeutic drug monitoring.

## Data Availability

No new data were created or analyzed in this study. Data sharing is not applicable to this article.

## Supplementary Materials

The following supporting information can be downloaded at: https://www.mdpi.com/article/10.3390/jof12080596/s1, Table S1: Specific reassessment priorities in rare filamentous fungal infections and disseminated endemic mycoses; Table S2: Potential quality indicators for AFSPs in therapeutic failure scenarios.
