# Supplementary material for: Therapeutic Failure in Invasive Fungal Infections: Beyond Antifungal Resistance—A Narrative Review
Source: J Fungi (Basel). 2026 Aug 11;12(8):596. doi: 10.3390/jof12080596 (PMC13514326; doi:10.3390/jof12080596)
Supplement: Supplementary file 1 [file jof-12-00596-s001.zip › jof-4477692-supplementary.pdf]

Table S1. Specific Reassessment Priorities in Rare Filamentous Fungal Infections and Disseminated Endemic Mycoses [4,5,42,95,136,138,159,160,170,177–179]

Clinical entity or group	Frequent determinants	Common clinical error	Reassessment priority	Initial guidance
Invasive fusariosis	Profound neutropenia; hematogenous dissemination; fungemia; skin lesions; variable antifungal susceptibility; insufficient immune recovery.	Escalating antifungal treatment without defining disease extent, species, antifungal susceptibility, or the potential for immune recovery.	Confirm identification and disease extent; assess antifungal susceptibility; and reassess neutropenia and cutaneous, pulmonary, or disseminated foci.	Individualize treatment; promote immune recovery when feasible; control localized foci; and seek expert input.
Scedosporiosis	Difficult identification; reduced antifungal susceptibility; pulmonary, osteoarticular, or CNS involvement; limited therapeutic evidence.	Treating the infection as an unspecified rare mold without differentiating the species or anatomical site.	Confirm the species, antifungal susceptibility, and disease extent; assess expected antifungal penetration into the affected site.	Tailor treatment to the species and anatomical site; consider surgery or drainage when appropriate; and seek expert multidisciplinary discussion.
Lomentosporiosis	Intrinsic resistance or markedly reduced susceptibility; disseminated disease; profound immunosuppression; few therapeutic alternatives.	Continuing empirical treatment without recognizing the characteristic resistance profile of *Lomentospora prolificans*.	Confirm identification and antifungal susceptibility; assess dissemination and the feasibility of source control.	Individualize salvage treatment; optimize source control and reversal of immunosuppression when feasible; and consult a specialized center.
Other rare or emerging molds	Inadequate resolution of conventional identification; unpredictable antifungal susceptibility; insufficient initial antifungal coverage; deep-seated foci; limited evidence.	Continuing empirical treatment without advanced identification or renewed mycological assessment.	Refer the isolate or specimen; consider an updated MALDI-TOF MS database, sequencing, or PCR according to availability.	Individualize treatment according to identification, antifungal susceptibility, anatomical site, and organism-specific evidence; prioritize source control.
Disseminated histoplasmosis	Delayed diagnosis; high fungal burden; advanced HIV infection; coinfections; limited access to antigen testing; impaired ITC absorption or drug–drug interactions.	Misdiagnosing the disease as TB or malignancy; failing to request antigen testing when available; or using ITC without reviewing antifungal exposure.	Confirm the diagnosis through antigen testing, culture, histopathology, or other available methods; search for coinfections; and reassess severity and immunosuppression.	Use L-AmB for severe disease; optimize transition to ITC; and review adherence, absorption, drug–drug interactions, and antigen trends.
Disseminated or meningeal coccidioidomycosis	Unrecognized geographic exposure; meningeal, bone, or skin involvement; immunosuppression; insufficient treatment duration.	Failing to integrate epidemiological exposure; underestimating CNS involvement or the need for prolonged treatment.	Confirm geographic exposure and disease extent; review serology, culture, or histopathology according to availability.	Select treatment according to severity and anatomical site; consider prolonged therapy and azole TDM when clinically relevant.

Disseminated paracoccidioidomycosis	Delayed diagnosis; advanced disease; pulmonary, mucocutaneous, lymph node, or adrenal involvement; barriers to diagnostic and therapeutic access.	Misdiagnosing the disease as TB, malignancy, or an inflammatory disorder; delaying mycological confirmation.	Confirm epidemiological exposure and systemic disease extent; assess pulmonary, mucosal, lymph node, and adrenal involvement.	Adapt treatment to disease severity; ensure an adequate treatment duration; and monitor complications and sequelae.
Talaromycosis	Advanced HIV infection; high fungal burden; delayed diagnosis outside endemic areas; nonspecific systemic manifestations.	Failing to recognize the epidemiological context; confusing the disease with TB, histoplasmosis, or another disseminated infection.	Review residence and travel history; confirm the diagnosis through culture, histopathology, or other available tests.	Initiate treatment according to disease severity; optimize management of immunosuppression; and search for coinfections.
Emergomycosis and other emerging endemic mycoses	Difficult diagnosis; limited availability of diagnostic tests; advanced immunosuppression; nonspecific manifestations; limited clinical experience.	Classifying the infection as an unspecified mycosis or initiating treatment without pursuing identification.	Expand the diagnostic workup using histopathology, culture, molecular methods, or referral to a reference laboratory.	Individualize treatment; seek expert advice; and integrate epidemiology and organism-specific evidence into decision-making.

Note: The proposed guidance does not replace disease-specific clinical guidelines or expert assessment. Treatment selection should be individualized according to organism identification, antifungal susceptibility, anatomical site, disease extent, host-related factors, and the available evidence.

CNS, central nervous system; HIV, human immunodeficiency virus; IFI, invasive fungal infection; ITC, itraconazole; L-AmB, liposomal amphotericin B; MALDI-TOF MS, matrix-assisted laser desorption/ionization time-of-flight mass spectrometry; PCR, polymerase chain reaction; TB, tuberculosis; TDM, therapeutic drug monitoring.

Table S2. Potential Quality Indicators for Antifungal Stewardship Programs in Antifungal Treatment-Failure Scenarios [14–18,21,24,30,35,49,62,100,143]

Domain	Potential indicator	Suggested operational definition	Data source	Usefulness for the AFSP
Clinical suspicion and diagnostic timeliness	Time from IFI suspicion to specimen collection	Interval between the first documented clinical suspicion of IFI and collection of the clinically relevant specimen.	Medical record; diagnostic orders; laboratory records; microbiology records.	Identifies delays in obtaining appropriate specimens before initiating or modifying antifungal treatment.
Initiation of active treatment	Time from suspicion to initiation of an active antifungal agent	Interval between documented suspicion of IFI and administration of an antifungal agent expected to be active against the probable clinical syndrome or identified pathogen.	Medical record; electronic prescribing system; pharmacy records; microbiology records.	Assesses therapeutic timeliness, particularly in candidemia, mucormycosis, and invasive aspergillosis.
Initial diagnostic assessment	Proportion of patients who undergo an appropriate initial diagnostic assessment	Percentage of patients who undergo the diagnostic tests predefined for the suspected syndrome and available within the institution or its referral network.	Medical record; laboratory records; radiology records; pathology records.	Identifies diagnostic gaps and supports the development or refinement of syndrome-specific institutional pathways.
Pathogen identification	Proportion of clinically relevant isolates identified to the species level	Percentage of clinically relevant isolates identified to the species level using conventional methods, MALDI-TOF MS, sequencing, or equivalent identification platforms.	Microbiology laboratory; reference laboratory.	Supports appropriate treatment selection and recognition of emerging species or species with unpredictable antifungal susceptibility.
Antifungal susceptibility testing	Proportion of eligible cases in which antifungal susceptibility testing is requested or referred	Percentage of clinically relevant isolates evaluated when the species, microbiological persistence, breakthrough IFI, poor response, or epidemiological context justifies antifungal susceptibility testing.	Microbiology laboratory; reference laboratory; medical record.	Assesses access to and appropriate use of antifungal susceptibility testing to guide treatment modification or de-escalation.
Reassessment after a poor response	Proportion of patients with documented structured reassessment	Percentage of patients with a suboptimal response for whom diagnostic certainty, host-related factors, pathogen characteristics, antifungal pharmacology, source control, and subsequent management are documented.	Medical record; infectious diseases service; AFSP records; multidisciplinary committee records.	Measures adherence to the reassessment algorithm and promotes transparent, traceable clinical decisions.
TDM in eligible patients	Proportion of eligible patients for whom TDM is requested	Percentage of patients receiving VRC, POS, ITC, or 5-FC who have a predefined clinical indication for TDM	Laboratory records; clinical pharmacy records; medical record.	Assesses optimization of antifungal exposure and prevention of subtherapeutic

		and undergo concentration measurement.		or potentially toxic concentrations.
Drug–drug interactions	Proportion of patients with a documented formal interaction review	Percentage of patients receiving systemic antifungal treatment who have a documented review of clinically relevant drug–drug interactions.	Clinical pharmacy records; medical record; electronic prescribing system.	Identifies preventable causes of inadequate antifungal exposure and improves treatment safety.
Source control in candidemia	Time to CVC assessment	Interval between the first positive blood culture and documentation of CVC removal, exchange, or clinically justified retention.	Medical record; microbiology records; infectious diseases service; procedure records.	Identifies delays in controlling a potential intravascular source of persistent candidemia.
Microbiological follow-up in candidemia	Proportion of patients with follow-up blood cultures	Percentage of patients with candidemia who undergo follow-up blood cultures until bloodstream clearance is documented.	Microbiology records; medical record.	Helps establish microbiological persistence, determine treatment duration, and identify the need to investigate deep-seated foci.
Investigation of deep-seated foci	Proportion of patients with persistent candidemia evaluated for deep-seated or metastatic foci	Percentage of patients with persistent or recurrent fungemia who undergo a targeted investigation for deep-seated or metastatic infection according to the clinical presentation.	Medical record; imaging records; echocardiography records; ophthalmology assessment; infectious diseases service.	Reduces erroneous attribution of microbiological persistence solely to antifungal resistance or insufficient pharmacological activity.
Surgical management in mucormycosis	Time from suspicion to surgical assessment	Interval between clinical, radiological, microbiological, or histopathological suspicion of mucormycosis and assessment by the relevant surgical team.	Medical record; consultation records; surgical records.	Assesses the timeliness of a potentially decisive source-control intervention.
Correction of predisposing factors in mucormycosis	Proportion of eligible patients with a documented targeted intervention	Percentage of patients with modifiable metabolic or immunological risk factors for whom a targeted corrective intervention is documented.	Medical record; laboratory records; endocrinology service; ICU records.	Assesses intervention on non-pharmacological determinants that may contribute to a poor response.
Documentation of treatment intensification	Proportion of treatment changes with an explicit clinical objective	Percentage of antifungal changes, combinations, or intensifications for which the rationale, therapeutic objective, reassessment time point, and exit or de-escalation criterion are documented.	Medical record; AFSP records; clinical pharmacy records.	Reduces unjustified treatment intensification and improves the traceability of complex therapeutic decisions.
De-escalation or discontinuation	Proportion of patients whose treatment is narrowed or discontinued when active IFI is not confirmed	Percentage of patients in whom the antifungal spectrum is narrowed or treatment is discontinued after active IFI is excluded, colonization is identified, or an alternative diagnosis is confirmed.	Medical record; pharmacy records; microbiology records; AFSP records.	Reduces avoidable toxicity, costs, and antifungal selective pressure.

Multidisciplinary discussion	Proportion of complex cases reviewed by a multidisciplinary team	Percentage of cases meeting locally predefined complexity criteria that are discussed by the relevant clinical, microbiological, pharmacological, surgical, or critical-care services.	AFSP meeting records; medical record; multidisciplinary meeting notes.	Assesses integration of care and coordination of diagnostic and therapeutic decisions.
Outcome of reassessment	Proportion of patients with an identified main determinant and a linked clinical action	Percentage of patients in whom at least one main determinant of the poor response is identified and a corresponding clinical action is documented.	Medical record; AFSP records; clinical audit.	Measures the ability of the reassessment process to translate diagnostic findings into a specific and evaluable intervention.

Note: Indicators should be predefined according to the clinical syndrome, adapted to local epidemiology and institutional capacity, and validated before being used for institutional comparisons or performance assessment. Compliance should not be interpreted in isolation or used as a substitute for individualized clinical assessment. Indicators may require access to institutional or referral-network resources and should therefore be interpreted within the context of locally available diagnostic and therapeutic capabilities.

5-FC, flucytosine; AFSP, antifungal stewardship program; CVC, central venous catheter; ICU, intensive care unit; IFI, invasive fungal infection; ITC, itraconazole; MALDI-TOF MS, matrix-assisted laser desorption/ionization time-of-flight mass spectrometry; POS, posaconazole; TDM, therapeutic drug monitoring; VRC, voriconazole.
